# Exploring semantic deep learning for building reliable and reusable one health knowledge from PubMed systematic reviews and veterinary clinical notes

**DOI:** 10.1186/s13326-019-0212-6

**Published:** 2019-11-12

**Authors:** Mercedes Arguello-Casteleiro, Robert Stevens, Julio Des-Diz, Chris Wroe, Maria Jesus Fernandez-Prieto, Nava Maroto, Diego Maseda-Fernandez, George Demetriou, Simon Peters, Peter-John M. Noble, Phil H. Jones, Jo Dukes-McEwan, Alan D. Radford, John Keane, Goran Nenadic

**Affiliations:** 1 School of Computer Science, University of Manchester, Manchester, UK; 2 Hospital do Salnés, Villagarcía de Arousa, Pontevedra, Spain; 3 BMJ, Tavistock Square, London, UK; 4 Salford Languages, University of Salford, Salford, UK; 5 Departamento de Lingüística Aplicada a la Ciencia y a la Tecnología, Universidad Politécnica de Madrid, Madrid, Spain; 6 Midcheshire Hospital Foundation Trust, NHS England, Crewe, UK; 7 School of Medical Sciences, University of Manchester, Manchester, UK; 8 School of Social Sciences, University of Manchester, Manchester, UK; 9 Small Animal Veterinary Surveillance Network, University of Liverpool, Liverpool, UK; 10 Small Animal Teaching Hospital, University of Liverpool, Liverpool, UK; 11 Manchester Institute of Biotechnology, University of Manchester, Manchester, UK; 12 Health eResearch Centre, University of Manchester, Manchester, UK

**Keywords:** Semantic deep learning, Ontology, Deep learning, CBOW, Skip-gram, One health, SNOMED CT, PubMed, Veterinary clinical narratives, Module extraction

## Abstract

**Background:**

Deep Learning opens up opportunities for routinely scanning large bodies of biomedical literature and clinical narratives to represent the meaning of biomedical and clinical terms. However, the validation and integration of this knowledge on a scale requires cross checking with ground truths (i.e. evidence-based resources) that are unavailable in an actionable or computable form. In this paper we explore how to turn information about diagnoses, prognoses, therapies and other clinical concepts into computable knowledge using free-text data about human and animal health. We used a Semantic Deep Learning approach that combines the Semantic Web technologies and Deep Learning to acquire and validate knowledge about 11 well-known medical conditions mined from two sets of unstructured free-text data: 300 K PubMed Systematic Review articles (the PMSB dataset) and 2.5 M veterinary clinical notes (the VetCN dataset). For each target condition we obtained 20 related clinical concepts using two deep learning methods applied separately on the two datasets, resulting in 880 term pairs (target term, candidate term). Each concept, represented by an n-gram, is mapped to UMLS using MetaMap; we also developed a bespoke method for mapping short forms (e.g. abbreviations and acronyms). Existing ontologies were used to formally represent associations. We also create ontological modules and illustrate how the extracted knowledge can be queried. The evaluation was performed using the content within BMJ Best Practice.

**Results:**

MetaMap achieves an F measure of 88% (precision 85%, recall 91%) when applied directly to the total of 613 unique candidate terms for the 880 term pairs. When the processing of short forms is included, MetaMap achieves an F measure of 94% (precision 92%, recall 96%). Validation of the term pairs with BMJ Best Practice yields precision between 98 and 99%.

**Conclusions:**

The Semantic Deep Learning approach can transform neural embeddings built from unstructured free-text data into reliable and reusable One Health knowledge using ontologies and content from BMJ Best Practice.

## Background

One Health is an approach to achieve better public health outcomes by combining efforts from different disciplines and domains [1]. It entails the recognition that the health of animals and the environment are essential for human health, and – specifically – that human medicine can benefit from veterinary medicine, as animals develop many of the same diseases as humans do [2]. Zoonotic infections and anti-microbial resistance are examples that have received much of the attention recently. A recent study by Stroud et al. [3] compiled several examples of One Health cases where human medicine can benefit from veterinary studies. However, while both biomedical and clinical knowledge about human and animal health are growing, they remain isolated silos. This paper investigates to what extent it is possible to acquire One Health knowledge from the evidence-based biomedical literature and veterinary clinical narratives.

We focus on using Semantic Deep Learning (SemDeep) [4, 5] – an emerging area combining the Semantic Web resources and technologies and Deep Learning for this task. Deep Learning is an area of machine learning that applies neural networks to “*learn representations of data with multiple levels of abstraction*” [6]. The Semantic Web embodies standards and tools for publishing and processing meta-data, with ontologies at its core. This paper proposes the use of ontologies as a way of specifying the meaning of semantically related terms derived from neural language models obtained via Deep Learning. We explore how to turn information about diagnoses, prognoses, therapies, as well as other clinical/healthcare entities into computable knowledge “*integrating individual clinical expertise and best external evidence*” [7].

There are two main challenges to achieving actionable or computable knowledge about human diseases or syndromes:

Multiple evidence-based resources – such as BMJ Best Practice [8], DynaMed Plus [9] or UptoDate [10] – are consulted by clinicians on a daily basis to assist clinical decision making at point-of-care. Healthcare professionals need to provide effective and safe patient care, and this also implies complying with Clinical Practice Guidelines (CPGs) like those provided by the UK National Institute for Health and Care Excellence (NICE) [11]. CPGs tend to be evidence-based and have been defined as “*systematically developed statements to assist practitioners and patient decisions about appropriate health care for specific circumstances*” [12]. Typically, evidence-based resources, such as the NICE CPGs or BMJ Best Practice, consist of human-readable text that is updated periodically and is intended only for expert-to-expert communication. A fundamental obstacle for achieving interoperability between evidence-based resources is lack of “grounding” or “normalisation” [13], i.e. the fact that biomedical/clinical terms appearing in the NICE CPGs or BMJ Best Practice are not mapped to specific terminological entries like the Unified Medical Language System (UMLS) [14] Metathesaurus. Normalisation may help with periodic updates of evidence-based resources from the biomedical literature, as PubMed/MEDLINE articles are indexed with Medical Subject Headings (MeSH) [15], which is included in the UMLS Metathesaurus. Unfortunately, annotating biomedical articles with MeSH terms is difficult and expensive [16] and normalisation of evidence-based resources is not being carried out.

World-leading terminologies, such as the Systematized Nomenclature of Medicine Clinical Terms (SNOMED CT) [17], are included within the UMLS Metathesaurus and provide multiple terms for expressing a biomedical concept. However, SNOMED CT does not contain formal or semi-formal descriptions that help understand how a disease develops and which treatment approaches can be considered. For example, medication statements that state which drugs treat a disease or syndrome are not included in SNOMED CT. Hence, even automatic acquisition of a partial set of SNOMED CT concepts relevant for a disease or syndrome (e.g. medications/drugs and clinical findings) remains an unmet need.

This paper investigates whether a SemDeep approach can help address both challenges. On the one hand, the neural language models can be applied to PubMed Systematic Reviews [17], which is a large collection of evidence-based articles (e.g. clinical trials, systematic reviews, and CPGs) available in PubMed/MEDLINE. Vector representations of the terms (“word embeddings” or “neural embeddings”) can be learnt from this unstructured text corpus, where semantically related terms will end up close in the representational space. On the other hand, if the terms that participate in the associations derived from these distributional similarities are normalised (i.e. terms with vector representations are mapped to UMLS Metathesaurus concepts and SNOMED CT concepts), that would allow: a) the expansion of the knowledge that exists in SNOMED CT; and b) automatic derivation of SNOMED CT subsets of semantically related concepts that can be reused and shared.

In this paper we focus on 11 well-known medical conditions that affect both humans and animals: heart failure, asthma, epilepsy, glaucoma, chronic kidney disease, osteoarthritis, anaemia, arthritis, diabetes mellitus, hypertension, and obesity. We adhere to “comparative/translational medicine” [18] and investigate the added value of One Health by combining: a) the Systematic Reviews Subset of PubMed, and b) a large set of veterinary clinical narratives collected by the Small Animal Veterinary Surveillance Network (SAVSNET) [19]. We rely on existing ontologies (lemon (Lexicon Model for Ontologies) [20] and OBAN (Open Biomedical AssociatioNs) [21]) to formally represent associations (semantically related term pairs) derived from neural embeddings. However, as Deep Learning algorithms have a “*black-box representation*” [22], their wider acceptance and adoption in the biomedical and clinical domain requires confidence and trust. To build such confidence (through transparency and interpretability), we use evidence-based resources (namely BMJ Best Practice) to verify if the associations (the semantic relatedness) captured by neural embeddings are reliable for human medicine. We note that clinicians consult multiple evidence-based resources, so BMJ Best Practice cannot be taken as the only gold standard that provides ground-truth. However, in practical terms, when there is a lack of external evidence from systematic research about the meaningful association of two terms (e.g. a medical condition and a treatment), the term pair should be considered as unrelated.

### Related work

Neural embeddings learnt from the biomedical literature or clinical narrative corpora have been widely used from many tasks, but they pose a challenge when measuring their quality. On the one hand, the biomedical/clinical domain requires background knowledge that makes crowd-worker evaluation (e.g. users of Amazon Mechanical Turk) unsuitable. On the other hand, there are no similarity and relatedness benchmarks developed for well-known medical conditions per se. Currently, there are four main standard benchmarks that are specific to the medical/clinical domain and suitable for a semantic similarity and relatedness task: Caviedes and Cimino [23] with 10 medical term pairs; Pedersen et al. [24] with 30 medical term pairs; Pakhomov et al. [25] with 101 clinical term pairs; and Pakhomov et al. [26] with 724 medical term pairs (the last two available at [27]). In total, these standard benchmarks provide less than 1 K term pairs. It should be noted that similarity and relatedness benchmarks were used to evaluate traditional distributional semantic models [28] – e.g. Latent Semantic Analysis (LSA) [29] or Latent Dirichlet Allocation (LDA) [30]. Faruqui et al. [31] emphasise that the lack of standard evaluation methods for neural embeddings was the trigger to create new benchmark datasets (e.g. Simlex-999 [32] and SimVerb-3500 [33] that are outside of the biomedical/clinical domain) and highlight that “*the use of word similarity tasks for evaluation of word vectors is not sustainable and calls for further research on evaluation methods*”.

A common characteristic of biomedical/clinical documents is that “*longer words and phrases are frequently mapped onto a shorter form such as abbreviations or acronyms for efficiency of communication*” [34]. For example, “heart failure” (long form) can appear as “HF” (short form). Another issue is that “*the number of abbreviations and the average number of definitions per abbreviation*” is ever growing [34]. For example, “HF” (short form) can have multiple meanings, and therefore, refer to multiple senses besides “heart failure”, such as “Hispanic female” or “high-fat” or “Hartree-Fock” or “hemofiltration”. Although short forms (abbreviations and acronyms) are present in UMLS, several studies [35–37] have shown UMLS to have shortcomings when mapping short forms to long forms. Sense inventories have been created such as SaRAD [38], ADAM [39], and more recently Allie [40] – in May 2018, Allie contained 840 K of short forms. These sense inventories and their algorithms assume that the short and long form co-occur in the biomedical literature, such as MEDLINE/PubMed abstracts; however, they rarely co-occur in clinical narratives [41, 42]. Sense inventories from clinical documents are fewer in number than sense inventories from the biomedical literature and contain fewer short forms. Wu et al. [43] highlight that “*accurate identification of clinical abbreviations is a challenging task and advanced abbreviation recognition modules are needed for existing clinical NLP systems*”. Dealing with short forms is therefore a challenge that requires an approach to deal with terms appearing in both biomedical and clinical documents.

The paper is organised as follows. In the next section, we present a SemDeep approach that builds on our previous work [44–47]. We first introduce the datasets, and then present the approach that deals with two NLP tasks: a semantic similarity and relatedness task, and a named-entity recognition (NER) task. As part of the SemDeep pipeline, we show how to reuse existing ontologies to formally represent the associations derived from neural embeddings in OWL. We also create ontological modules with the SNOMED CT ontology and illustrate how to query the extracted knowledge using the SPARQL query language [48], which exploit the underlying ontological representations and can be executed using Jena ARQ [49].

## Materials and methods

### Data

The PubMed Systematic Reviews dataset (the PMSB dataset): we downloaded the MEDLINE/PubMed baseline files for 2015 and up-to-date files through 8th June 2016. Applying the PubMed Systematic Reviews filter [17], a subset of 301,201 PubMed publications published between 2000 and 2016 was obtained. We extracted titles and available abstracts. We note that this dataset was also used in our previous study on sepsis [46].

The SAVSNET dataset (the VetCN dataset): a collection of 2,465,420 de-identified and non-empty veterinary clinical narratives was obtained from SAVSNET on 20th October 2017. SAVSNET is an initiative by the British Small Animal Veterinary Association and the University of Liverpool that collects free-text consultations notes across around 500 UK veterinary premises in real-time.

We used the UMLS Metathesaurus with close to 3.7 M biomedical/clinical concepts with 203 sources contributing to concept names (as of May 2018), including SNOMED CT and the Veterinary Extension for SNOMED CT (VetSCT) [50]. We used MetaMap 2016v2 (released September 2016) and the SNOMED CT version released in January 2017 in OWL to maximise compatibility among versions. When using the UMLS Terminology Services [14] to access the UMLS Metathesaurus online, we select the UMLS2016AB version. As we cannot release a subset of the UMLS Metathesaurus or SNOMED CT, to replicate the results obtained with our method it is necessary to use the UMLS API [51] and the OWL API [52].

### SemDeep pipeline

Below we describe each step of the pipeline intended to acquire and validate knowledge about medical conditions from the unstructured text datasets.

#### Step 1: computing n-grams from unstructured text

We employed word2phrase within the word2vec software package [53] to compute n-grams from the unstructured text datasets (e.g. PMSB or VetCN). It should be noted that text within consultation notes (e.g. “today pain++”) contains a significant number of misspellings, local abbreviations and short forms, and lacks the grammatical correctness found within biomedical literature. After applying word2phrase, the character “_” appears within tokens that co-occur repeatedly together (e.g. “heart_failure” is a bigram with two tokens). Each n-gram has a frequency count. We note that an n-gram captures words or tokens that appear together in a textual corpus with a certain frequency. Therefore, an n-gram can capture words and phrases as well as combinations of tokens that may not correspond exactly to meaningful unit when looked in isolation phrase, e.g. “(COPD)_is_a”.

#### Step 2: creation of neural embeddings

We create neural embeddings with CBOW (Continuous Bag-of-Words) and Skip-gram [54] using the word2vec software. The input for CBOW and Skip-gram is the unstructured text with n-grams, where the character “_” typically denotes the presence of an n-gram of size greater than one (e.g. a bigram or a trigram). The output for CBOW and Skip-gram is typically: 1) a lexicon (i.e. a list of n-grams) that is present in the unstructured text and for which the vector representations have been learnt; and 2) a binary file that contains neural embeddings, i.e. real-number representations for the terms in the lexicon. When producing neural embeddings, there are a small number of hyperparameters that need to be tuned – we used the hyperparameters configuration described in our previous work [55].

#### Step 3: obtaining term pairs for the semantic similarity and relatedness task

Taking the vector for a specific target term (e.g. a disease or syndrome) and applying the cosine similarity, a list of top ranked terms (highest cosine values) can be obtained from the created neural embeddings. These top ranked terms are candidate terms, i.e. terms that need to be judged as semantically similar or related to the target term. Some authors agree that “*semantic similarity represents a special case of semantic relatedness*” [24]; Hill et al. [32] interpret “relatedness” as “association”, where the strongest similarity relation is synonymy; this interpretation is applied in this study.

*Target terms selection*: to choose the target terms, we first select the n-grams that pass the threshold of 1 K frequency count and MetaMap has assigned them UMLS Metathesaurus concepts with the Semantic Type “T047|Disease or Syndrome”. The final selection of target terms needs to be done manually as: a) MetaMap may erroneously assign a UMLS Metathesaurus concept to an n-gram, particularly if the n-gram is a short form such as “HF”; and b) the well-known medical conditions selected as target terms should be covered by BMJ Best Practice – the preferred gold standard in this study.

*Number of top ranked candidate terms per target term*: to the best of our knowledge, no published study justifies the number of terms selected with the highest cosine value. Different studies used different numbers: from three [56], ten [57, 58], 40 [59] to a range of numbers (e.g. 5, 10, 20, 40, and 100) [60]. We limit the list of candidate terms to the 20 n-grams with the highest cosine value.

#### Step 4: named entity recognition

Named Entity Recognition (NER) consists of “*identifying specific words or phrases (‘entities’) and categorizing them*” [61]. We use MetaMap to categorise the n-grams into one or more of 133 broad categories (Semantic Types) from the UMLS Metathesaurus. To determine if MetaMap supplies a correct CUI for an n-gram, detailed guidelines were developed that intend to favour compatibility with the SNOMED CT Compositional Grammar [62]:

- Single Map (SM) – MetaMap provides a single CUI that captures the full meaning of the n-gram. This case corresponds to “Simple Expression” [62], i.e. a single concept identifier. For example, the n-gram “septic_shock” is mapped only to UMLS CUI = C0036983.
- Multiple Maps (MM) – MetaMap provides multiple CUIs that capture the meaning of the n-gram, and one or more focus concepts may appear among the CUIs provided. This case may also correspond to “Simple Expression” [62], although it more often corresponds to “Expression with Refinements” or “Multiple Focus Concepts” [62]. Selection of focus concept(s) is guided by six principles described in the Additional file 2.
- Incorrectly Mapped (IM) – MetaMap provides one or more CUIs, however, none captures the meaning of the n-gram. For example, the n-gram “HF” is not mapped to “C0018801|Failure, Heart (Heart failure)”.
- Not Mapped (NM) – MetaMap does not provide any CUI. For example, the n-gram “HFpEF” is not mapped to “C3889077|Heart failure with preserved ejection fraction”.

Three domain experts (two biomedical terminologists and a medical consultant) inspected the results of MetaMap considering the above-mentioned guidelines and assigned to each candidate term (n-gram) one of the above values {SM, MM, IM, NM}. Beside the candidate term (n-gram), we also provide the target terms (n-grams) in lower case to provide local context. When an n-gram is incorrectly mapped or not mapped, a CUI for the n-gram is manually assigned. On the one hand, the guidelines presented aim to reduce the number of CUIs assigned to each n-gram. On the other hand, the n-grams are the result of statistical NLP and may contain short forms, and therefore, decomposing the n-grams into lexico-semantic units of meaning has proved demanding in our previous work [45–47] (even with years of experience doing clinical coding). Hence, the three domain experts worked together to identify the minimal semantic constituents of the n-grams according to the guidelines, i.e. determining systematically the focus concept(s).

### Dealing with short forms

We created a short form detector to identify n-grams with or without one or more clinically meaningful short forms. The detector is based on a hybrid approach: it contains if-then-else heuristic rules and utilises two lists of terms, i.e. a list of measurement units compiled from three resources [63–65] and the rank frequency list of the British National Corpus of written and spoken English [66] with 7726 words. The underlying assumption is that a measurement unit can have one or more short forms. For example, “mmHg” is a short form for the long form “millimetre of mercury”.

**Fig. 1:**
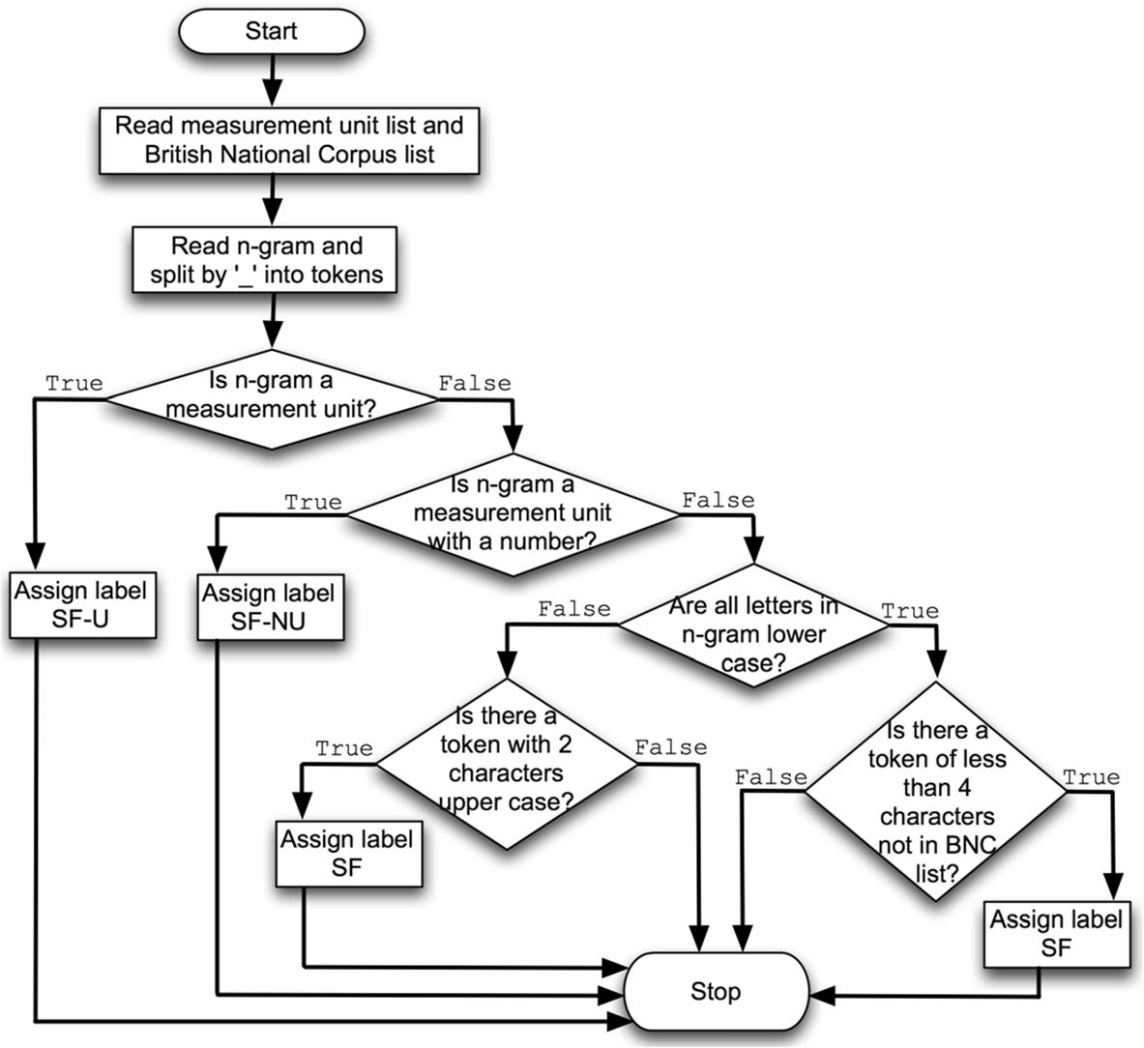
*Flowchart of the short form detector introduced* – a diagrammatic representation outlining how the short form detector assigns the labels {SF-U, SF-NU, SF}. If no label is assigned, this means that the n-gram has no clinically meaningful short form(s)

Figure 1 depicts a flowchart outlining how the short form detector that assigns one of the following labels to an n-gram:

- “SF-U” when an n-gram contains a unit of measurement. The n-gram is mapped to the UMLS Metathesaurus concept “C1519795|Unit of Measure”.
- “SF-NU” when an n-gram contains a number with a unit of measurement. The n-gram is mapped to the UMLS Metathesaurus concept “C0242485|Measurement”.
- “SF” when an n-gram contains a short form token that is not a measurement unit or a measurement unit and a number.
- No label when an n-gram does not contain a short form.

For those n-grams with a short form that is not a measurement unit or a measurement unit and a number, the domain experts manually utilised Allie as the preferred sense inventory, for expanding short forms into long forms. The reasons for using Allie are: a) it contains a much larger number of short forms than the UMLS SPECIALIST Lexicon; b) it has long forms for a short form ranked based on appearance frequency in PubMed/MEDLINE abstracts; and c) for each long form the research area and co-occurring abbreviations are provided, thus aiding disambiguation.

The short form detector can make two errors, and the domain experts will assign the following labels to an n-gram:

- “SF-I” denotes that a short form identified in an n-gram was assessed as not clinically meaningful, i.e. “incorrect”.
- “SF-NF” denotes that a clinically meaningful short form was not identified in an n-gram, i.e. “not found”.

### Experiment set-up and performance measures

We investigate the impact of the short forms on the performance of MetaMap via two experiments:

- Experiment 1 (EXP1): we expose the candidate terms directly to MetaMap, i.e. the lists with the 20 top-ranked n-grams (i.e. the 20 n-grams with the highest cosine value) are taken as input for MetaMap.
- Experiment 2 (EXP2): we expose firstly the candidate terms (n-grams) to the short form detector described above, and for those n-grams with one or more short forms, we expand each short form into the corresponding long form by utilising Allie. Once the short forms are replaced with the long forms, we take the modified/expanded candidate terms (n-grams) as input for MetaMap.

For both experiments, we assume that the candidate terms are biomedical or clinical terms, and thus, there should be no True Negatives (TNs). We use the conventional evaluation measures of Precision, Recall, and F measure [67] to calculate the MetaMap performance. As Pratt and Yetisgen-Yildiz [68], we have weaker MetaMap precision and recall where exact matches (typically Single Map) and partial matches (typically Multiple Maps) are equally counted, i.e. SM and MM are considered as True Positives (TPs). An Incorrectly Mapped (IM) is interpreted as False Positive (FP). A Not Mapped (NM) is interpreted as a False Negative (FN). Hence, we calculate precision as TP/(TP + FP) [67] and recall as TP/(TP + FN) [67]. To calculate F measure, we use equal weighting of precision and recall, calculating F measure as (2 x Precision x Recall)/(Recall + Precision) [67]. We compute precision, recall, and F measure for each well-known medical condition under study (i.e. target term), and then, average each evaluation measure over all to obtain an overall measure of performance (a.k.a. macro-averaging) [67]. We also report micro-averaging, i.e. making a single contingency table for all data [67] – all lists of the 20 n-grams with highest cosine value that are the input to MetaMap.

Following Smucker et al. [69], we measure statistical significance of the difference in the mean average precision, recall, and F measure to judge if there is a statistically significant improvement in performance for EXP2 (short form detection and expansion into long form before applying MetaMap to the unique candidate terms) when compared with the performance for EXP1 (applying MetaMap to the unique candidate terms). We use the Student’s paired t-test [70] as implemented in scikit-learn [71] to compare performance of EXP1 and EXP2.

All unique candidate terms (n-grams) are exposed to the short form detector. We also compute precision, recall, and F measure for the short form detector considering all candidate terms (micro-averaging) and the capability of the short form detector to identify n-grams with or without one or more clinically meaningful short forms.

#### Step 5: validation of the term pairs mapped to UMLS Metathesaurus concept pairs using BMJ best practice

This step validates the candidate terms obtained in Step 3 for the semantic similarity and relatedness task using BMJ Best Practice, which covers prevention, diagnosis, treatment and prognosis for well-known medical conditions. It contains “top down” knowledge manually extracted by medical experts and is based both on the latest clinical practice guidelines and underlying research evidence. Hence, BMJ Best Practice can be considered an evidence-based gold standard, supporting frontline clinicians.

The validation involves four domain experts. The three experts from the previous step (two terminologists and a medical consultant) together validate the concept pairs considering BMJ Best Practice and external resources (whenever necessary). A health informatician – who works with the content of BMJ Best Practice – contributes to the final stages of validation of the concept pairs and (additionally) provides feedback to the editors of BMJ Best Practice. To avoid bias, the domain experts validate the concept pairs without knowing: a) the dataset from which the concept pairs were derived; and b) the neural language models applied to the dataset. To avoid further hints about the underlying dataset, the target terms (n-grams) are presented in lower case.

The outcome of this step is a set of 3-tuples (target concept, candidate concept, validation label). The target and candidate concepts are UMLS Metathesaurus concepts representing the focus concept(s) of the n-grams for the term pairs (target term, candidate term). The validation label indicates how the matching between a candidate concept and a term from BMJ Best Practice is performed and reflects the amount of domain knowledge required to perform such a match. We distinguish six cases when matching a candidate concept name from the UMLS Metathesaurus to a term appearing in BMJ Best Practice:

1. *Itself* – a candidate term may have as its focus concept(s) the same UMLS Metathesaurus concept as the target term (i.e. the well-known medical condition), and therefore, they will not be matched to terms appearing in BMJ Best Practice. This case denotes synonymy (i.e. the strongest similarity relation).
2. *Relatedness by exact/approximate match* – candidate concept names as they appear in the UMLS Metathesaurus may match exactly or approximately terms appearing in BMJ Best Practice, where the biomedical/clinical meaning is the same. This typically denotes a similarity relation between the candidate concept and the BMJ term. This case can be interpreted as “normalising” BMJ Best Practice terms. For example, “pyrexic” (adjective) when used to refer to patients with “pyrexia” (noun) can be interpreted as “pyrexia”. From a linguistic point of view, “pyrexic” is a morphological variant of “pyrexia”, and thus, this is an example of an approximate match where the biomedical/clinical meaning is the same. Some “implicit knowledge” may be needed for this case.
3. *Relatedness by inexact match (hypernym/hyponym) or (hyponym/hypernym)* – candidate concept names as they appear in the UMLS Metathesaurus may have is-a relations with the terms appearing in BMJ Best Practice or vice versa. An is-a relation is also similar to a hypernym/hyponym relation or generalisation/specialisation relation and it denotes a similarity relationship. For example, “bacterial sepsis” is-a type of “sepsis”, where “bacterial sepsis” is the hyponym (specialisation) and “sepsis” is the hypernym (generalisation). Hypernym/hyponym relations can be used to build semantic taxonomies (a.k.a. hierarchies). Some “implicit knowledge” may be needed for this case.
4. *Relatedness by inexact match (background knowledge)* – candidate concept names as they appear in the UMLS Metathesaurus may have similarity (similar meaning or is-a relations) or relatedness (association) relations with the terms appearing in BMJ Best Practice, although the relations are not obvious for someone lacking biomedical/clinical knowledge. To make the “implicit knowledge” explicit, one or more excerpts of external evidence from systematic research or terminological resources are provided. This case may apply transitivity, i.e. “if A is related to B and B is related to C, then A is related to C”. Therefore, by making known one or more terms (call them B) it is feasible to make transparent how a UMLS Metathesaurus concept A related to a term C appearing in BMJ Best Practice. In other words, by making B known, and how A and C relate to B, implicit knowledge becomes “explicit”.
5. *Unrelated: not clinically meaningful* – An n-gram can capture combinations of words or tokens that can be mapped to a focus UMLS Metathesaurus concept(s), although it may not be interpreted per se as clinically meaningful in connection with a given medical condition. For example, “guided” is a unigram for which alternative UMLS Metathesaurus concepts are available to represent multiple meanings or senses: “C0181090|Guide (Professional guide)”; “C0302614|Guide (Guide device)”; and “C1706050|Guide (Guide Device Component)”. However, for “sepsis”, any sense for the n-gram “guided” per se is not clinically meaningful.
6. *Unrelated: excluded* – candidate concept names as they appear in the UMLS Metathesaurus may not have up-to-date clinically meaningful association (relatedness) relations with the terms appearing in BMJ Best Practice, even if there is implicit knowledge that can justify the association. A typical example of this case are treatments or therapies that became known as ineffective or have adverse effects. For example: “*The only novel anti-sepsis agent to successfully complete a phase 3 sepsis trial, human recombinant activated protein C, was recently taken off the market after a follow up placebo-controlled trial (PROWESS SHOCK) failed to replicate the results of the initial registration trial (PROWESS) performed 10 yr earlier.*” [72]

The process of assigning the six validation labels introduced above is iterative. A focus concept for a candidate term and a term from BMJ Best Practice may have been allocated the “Relatedness by Inexact match (background knowledge)” label to indicate that one or more excerpts of external evidence from systematic research or terminological resources are needed to establish “relatedness”. However, a closer inspection of the “implicit knowledge” that has become explicit may change the label into: “Relatedness by exact/approximate match”, or “Relatedness by Inexact match (hypernym/hyponym) or (hyponym/hypernym)”, or “Unrelated: Excluded”. The cases under the “Relatedness by Inexact match (background knowledge)” label may be further refined, for example, by considering the relationship between cause and effect.

The six validation labels introduced can be used for calculating precision by considering: a) the last two “unrelated” labels representing False Positives (FPs); and b) the “itself” and the labels starting with “Relatedness by” representing True Positives (TPs).

#### Step 6: formal representation of the knowledge acquired and validated

**Fig. 2:**
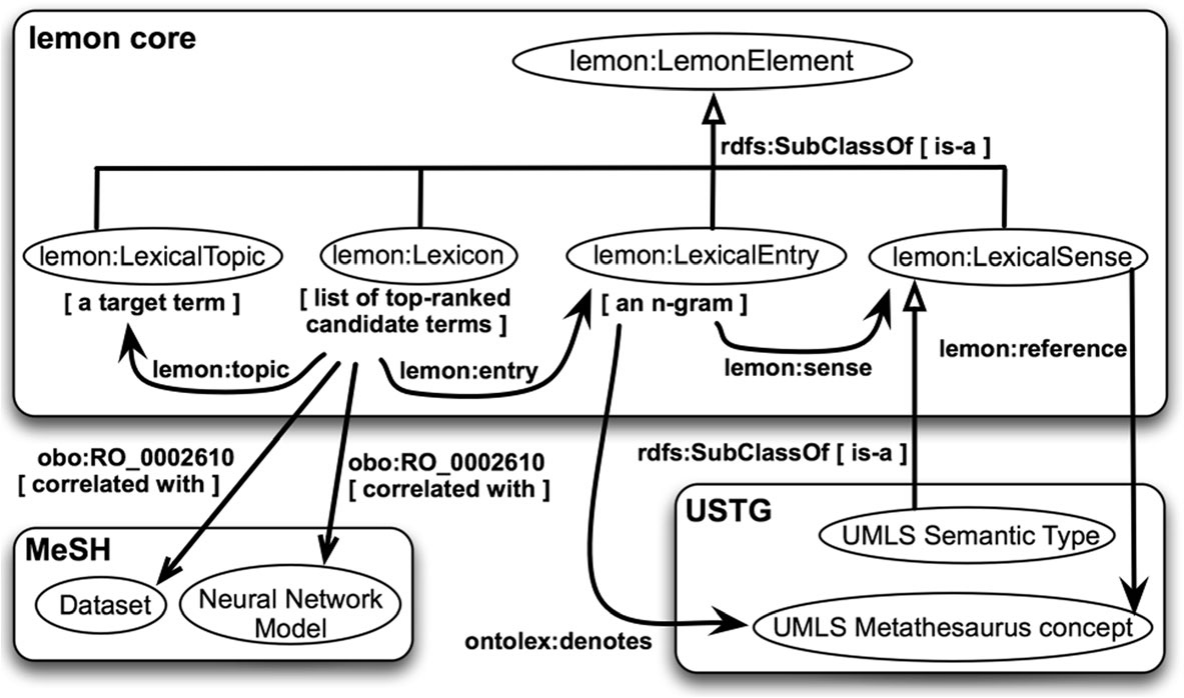
*Overview of the extended version of the lemon core ontology (called here the lemonEXT) used for this study*

**Fig. 3:**
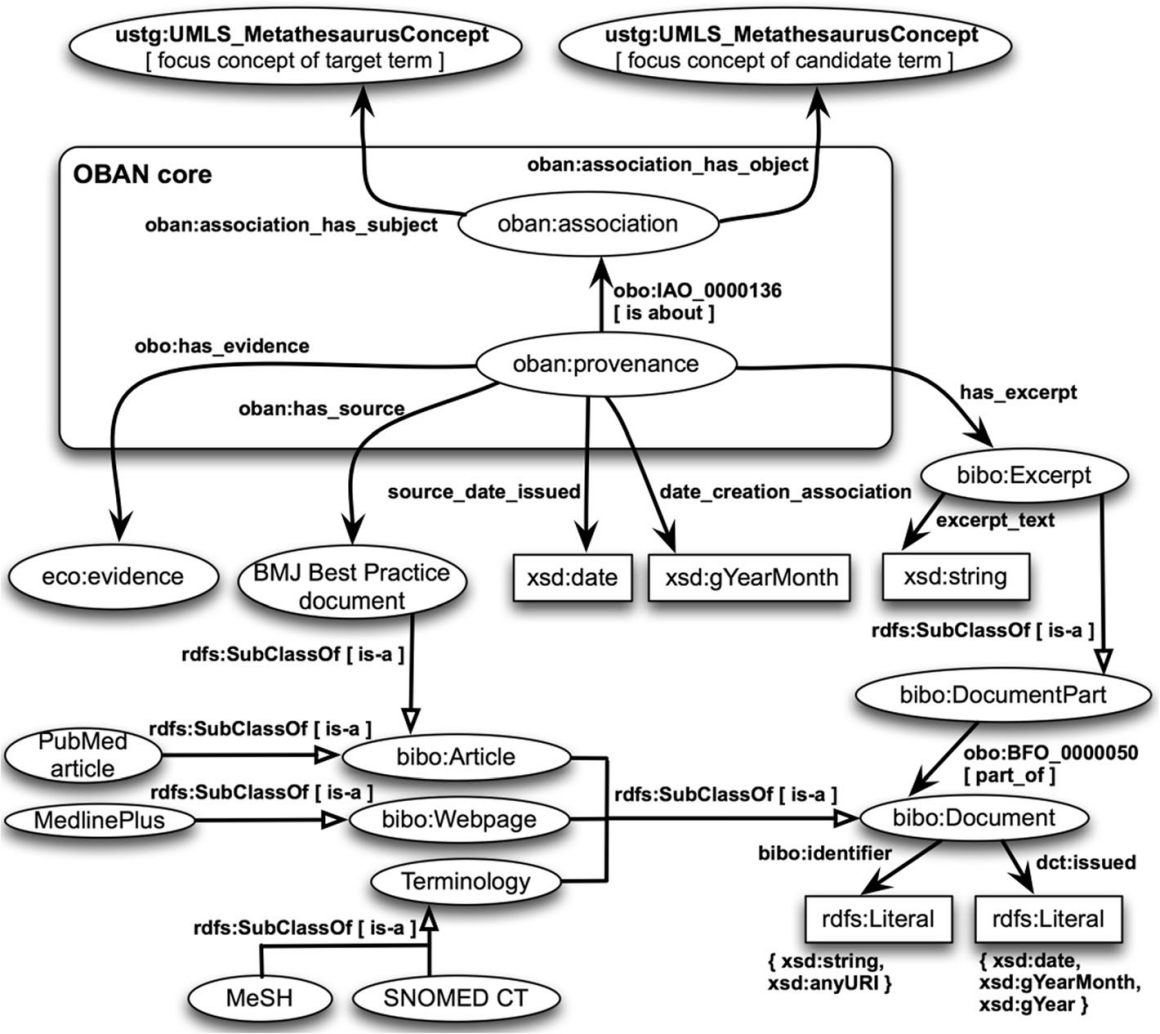
*Overview of the modified version of the OBAN core ontology (called here the OBANmod) used for this study*

We use OWL-DL to formally represent concept names, concept expressions, and terminological axioms. Figures 2 and 3 overview the two core ontologies that will be populated, i.e. the extended lemon core ontology (called here the lemonEXT) [73] and the modified OBAN core ontology (called here the OBANmod) [74]. Both core ontologies reused the USTG (UMLS Semantic Types and Groups) core ontology in OWL-DL that we created programmatically and utilised in [45, 46]. The USTG core ontology represents in OWL the information publically available at [75].

The USTG core ontology represents formally the UMLS Semantic Types and Groups as well as the part-whole relations among them by reusing the OWL object properties “part_of” (obo:BFO_0000050) and “has_part” (obo:BFO_0000051) from the Basic Formal Ontology (BFO) [76]. The USTG core ontology also contains the UMLS Metathesaurus concept, an OWL class we created that can have as a subclass any Metathesaurus concept from the UMLS. A new addition to the USTG core ontology is the OWL annotation property “hasDbXrefInSCT” to create annotation assertion axioms that act as cross-reference between the UMLS Metathesaurus and SNOMED CT. The annotation property “hasDbXrefInSCT” is a sub-annotation property of the annotation property “database_cross_reference” (oboInOwl:hasDbXref) from the oboInOwl meta-model [77]. The USTG core ontology has a total of 593 axioms (class count: 151; individual count: 0) and its Description Logic (DL) expressivity is ALEI.

Table 1 shows the axiom patterns in Manchester OWL Syntax [78] for populating programmatically the main OWL Classes of lemonEXT and OBANmod core ontologies. In this study, a pattern (a.k.a. axiom pattern) can represent a set of OWL axioms.

**Table 1:** *The axiom patterns in Manchester OWL Syntax for populating the main classes of the two core ontologies:* the extended lemon core ontology (lemonEXT); and the modified OBAN core ontology (OBAMmod). Each axiom pattern in the second column contains variables, which can be easily identified as they start with the character “?”. The third column exemplifies OWL individuals that are the result of populating the axiom patterns introduced in the second column

Brief description of axiom pattern	Axiom pattern in Manchester OWL Syntax	Example of populating the Axiom pattern in Manchester OWL Syntax
Individual of the OWL class lemon:LexicalTopic	Individual:? x Annotations: label? lbx@en Types: ‘Lexical topic’	Individual: heart_failure Annotations: label “heart_failure”@en Types: ‘Lexical topic’
Individual of the OWL class lemon:LexicalEntry	Individual:? y Annotations: label? lby@en Types: ‘Lexical entry’ Facts: denotes? CUI	Individual: beta-blockers Annotations: label “beta-blockers”@en Types: ‘Lexical entry’ Facts: denotes ‘Adrenergic beta-Antagonists’
Individual of the OWL class lemon:Lexicon	Individual:? z Annotations: label? lbz@en Types: Lexicon Facts: ‘correlated with’? dataset, ‘correlated with’? model, entry? y1, … … … entry? y20	Individual: PMSB_CBOW_heart_failure Annotations: label “PMSB_CBOW_heart_failure”@en Types: Lexicon Facts: 'correlated with’ ‘PMSB dataset’, 'correlated with’ CBOW, entry beta-blockers, … … … entry ‘cardiac_resynchronization_therapy_(CRT)’
Individual of the OWL class oban:association	Individual:? Cpair Annotations: rdfs:label? lbCpair@en Types: association Facts: oban:association_has_object? CUI1, oban:association_has_subject? CUI2	Individual: ‘(C0017601,C0020581)’ Annotations: label “(C0017601,C0020581)”@en Types: association Facts: 'association has object’ Hyphema, 'association has subject’ Glaucoma
Individual of the OWL class oban: provenance	Individual:? ECpair Annotations: rdfs:label? lbECpair@en Types: provenance Facts: 'has source’? BMJdoc, 'has excerpt’? BMJterm, 'has evidence’? lbEvidence, 'has excerpt’? EEexcerpt, 'is about’? Cpair, 'date creation association’? d1^^string, 'source date issued’? d2^^string	Individual: ‘(C0017601,C0020581,Relatedness by inexact match (background knowledge))’ Annotations: label “(C0017601,C0020581,Relatedness by inexact match (background knowledge))”@en Types: provenance Facts: 'has source’ ‘BMJ Best Practice: Open-angle glaucoma’, 'has excerpt’ trabeculotomy, 'has evidence’ ‘Relatedness by inexact match (background knowledge)’, 'has excerpt’ EEexcerpt_70, 'is about’ ‘(C0017601,C0020581)’, 'date creation association’ “May-2018″^^string, 'source date issued’ “09-Dec-2016″^^string

### The extended lemon core ontology (lemonEXT)

In Fig. 2, the concept “Lexical Topic” represents the target term (i.e. an n-gram corresponding to a given medical condition); the concept “Lexicon” represents the lexicon, i.e. a list of the 20 top-ranked terms (n-grams with the highest cosine value for the target term) obtained with CBOW and Skip-gram; and the concept “Lexical Entry” represents an n-gram in the lexicon. We reuse two concepts from MeSH: “D064886|Data Set” and “D016571|Neural Networks (Computer)”. The latter is a MeSH heading that has the entry term “Neural Network Models”, which is the term that appears in Fig. 2. The two MeSH concepts are represented as OWL classes and they are connected with the lemon concept “Lexicon” by reusing the OWL object property “correlated with” (obo:RO_0002610) from the Relations Ontology (RO) [79] .

As depicted in Fig. 2, the concept “Lexical entry” from lemon is connected to the UMLS Metathesaurus concept from the USTG core ontology with the OWL object property “denotes” from the Ontology Lexicon (Ontolex) ontology [80]. We made the OWL Class “Lexical sense” from lemon a superclass of the OWL Class UMLS Semantic Type from the USTG core ontology. As one or more UMLS Metathesaurus concept(s) is the focus concept(s) for each n-gram, one or more UMLS Metathesaurus concept(s) from the UTSG ontology captures the senses or meanings of a lexical entry. Taking into account the UMLS Semantic Type(s) assigned to each UMLS Metathesaurus concept, it is possible to categorised the n-grams based on the OWL class descriptions within the USTG ontology – e.g. “C0036983|Septic Shock” is a subclass of “T046|Pathologic Function” – and therefore, we follow the “semantics by reference” principle from [81] that says: “*the expressivity and the granularity at which the meaning of words can be expressed depend on the meaning distinctions made in the ontology*”.

The lemonEXT core ontology has a total of 643 axioms (class count: 158; individual count: 0) and its DL expressivity is ALEI. Once the lemonEXT is populated, it is possible to create SPARQL SELECT queries retrieving for each target term (i.e. n-gram) the candidate concepts, i.e. the UMLS Metathesaurus concepts that are the focus concepts of candidate terms (n-grams). We built three queries (see the Additional file 4 for details) that retrieve candidate concepts based on few UMLS Semantic Types and a UMLS Semantic Group. The UMLS Semantic Types and Group chosen intend to bring forward candidate concepts related to the diagnoses and management of a given medical condition (i.e. the target term):

- The UMLS Semantic Types “T059|Laboratory Procedure”; “T060|Diagnostic Procedure”; and “T061|Therapeutic or Preventive Procedure”. These are the three subtypes of the UMLS Semantic Type “T058|Health Care Activity” [82]. We call the query q1 and it utilises these four UMLS Semantic Types.
- The UMLS Semantic Types “T034|Laboratory or Test Result”; and “T184|Sign or Symptom”. These are the two subtypes of the UMLS Semantic Type “T033|Finding” [82]. We call the query q2 and it utilises these three UMLS Semantic Types.
- The UMLS Semantic Group “Chemicals & Drugs” (a.k.a. CHEM) that contains UMLS Semantic Types that are chemicals taking into account their structural and functional perspective [82]. Some of the UMLS Metathesaurus concepts belonging to CHEM are typically drug treatments (medications). We call the query q3 and it utilises the Semantic Group CHEM.

### The modified version of the OBAN core ontology (OBANmod)

An OBAN association relates biomedical entities (e.g. X, Y) “without enforcing directionality on the link” [21] (i.e. “X is associated with Y” or “Y is associated with X”) and separating the association between entities from its provenance [21]. Although we can safely state that there is a clinically meaningful association for the term pair (heart failure, pulmonary edema) it does not mean that all patients with “heart failure” will also have “pulmonary edema”. Instead, we should interpret the term pair (heart failure, pulmonary edema) as a “sometimes true” association relationship [21] that can be represented in OWL as an OBAN association.

Figure 3 presents a modified version of the OBAN core ontology for this study considering:

1. The OBAN association is between two UMLS Metathesaurus concepts represented as OWL Classes as in [21], and we also use punning. It should be noted that OWL 2 DL relaxes the separation between classes and individuals.
2. The validations labels introduced in Step 5 are represented as OWL Classes that have as super-classes OWL Classes from the Evidence and Conclusion Ontology (ECO) [83] (release of 2018-04-06). A total of five OWL Classes from ECO have been reused as well as the subclass axioms for them in the ECO.
3. The provenance that validates the “sometimes true” association relationship is BMJ Best Practice. To represent a document from BMJ Best Practice, we first reuse the OWL Class “Document” from the Bibliographic Ontology Specification ontology (BIBO) [84], and then, create a new subclass with the name “BMJ Best Practice document”. Therefore, “BMJ Best Practice for chronic congestive heart failure” [85] will be an OWL instance of the OWL Class “BMJ Best Practice document”.
4. Excerpts from biomedical or clinical resources are needed for this study, such as: 1) the term(s) appearing in BMJ Practice that are key when validating the concept pairs; or 2) additional information that make the “implicit knowledge” explicit, such as excerpts from the scientific literature (e.g. PubMed articles or MedlinePlus [86] Webpages) or from terminologies like SNOMED CT [62]. To represent an excerpt, we reuse the OWL Class “Excerpt” from the BIBO. In the BIBO, an “Excerpt” is part of a “Document”. We create the annotation property “excerpt_text” to store terms from BMJ Best Practice or lines of text that are considered pertinent when making the “implicit knowledge” explicit. Therefore, in this study, every OBAN association will have at least one instance of the OWL Class “Excerpt”, i.e. BMJ term(s) that are key to validate the focus concept pairs for the term pairs.
5. The replacement and modification of OWL data properties from OBAN to better fit the current study: a) the OWL data property “date_creation_association” where we store the month and year when the association was validated; b) the OWL data property “source_date_issued” where we store the last update of BMJ Best Practice document used to validate the association; and c) relax the xsd:dateTime declarations from OBAN as sometimes it proves difficult to trace the publication date for a PubMed paper or the release day for a terminological resource.

The OBANmod core ontology has a total of 779 axioms (class count: 181; individual count: 6) and its DL expressivity is ALEHI (D). Once the OBANmod is populated, it is possible to refine the SPARQL queries q1 to q3 into q1V to q3V (see the Additional file 4 for details), where the candidate concepts (i.e. UMLS Metathesaurus concepts that are the focus concepts for the candidate terms) should have an up-to-date clinically meaningful association to the target concepts (the selected diseases or syndromes) according to BMJ Best Practice (i.e. human medicine), and thus, the UMLS Metathesaurus concept pairs should have the validation labels introduced in Step 5 starting with “Relatedness by” or the validation label “Itself”.

### Extracting locality-based modules

Our aim is the extraction of locality-based modules from the SNOMED CT ontology that are: 1) much smaller in size (i.e. number of axioms) than the SNOMED CT ontology; 2) as specific as possible for a given medical condition while being logically sound according to OWL-DL; and 3) can be reused and shared among organisations.

We extract a locality-based module (a.k.a. upper module) [87] per target term using as signature all the SNOMED CT concept identifiers mapped to UMLS CUI pairs validated with BMJ Best Practice content. We use the reasoner FaCT++ [88] for the method ModuleType BOT in the OWL API [52] as we did in [47]. A DL reasoner, like FaCT++, can calculate inferred information (e.g. inferred subsumption hierarchy) from the asserted information, i.e. the axioms within an ontology. A locality-based module contains at least all the (entailed) super-classes of an OWL class included in the signature [87] as well as all axioms relevant to the meaning of the OWL Classes in the signature. A SNOMED CT concept may have one or more attribute-value pairs [62], where the value of the pair is typically another SNOMED CT concept. Attribute-value pairs are considered relevant to the meaning of a SNOMED CT concept.

A locality-based module keeps the SNOMED CT top-level hierarchies for the OWL Class extracted, which is expected by the clinicians, and is likely to be smaller than the SNOMED CT ontology. The SNOMED CT ontology corresponding to the January 2017 release contains a total of 1.5 M axioms (Class count: 325 K; individual count: 0; Object property count: 80; SubClassOf axioms count: 246 K; and EquivalentClasses axioms count: 79 K) and its DL expressivity is ALER.

Multiple SPARQL queries can be built seeking to gain insights into diseases and syndromes of significance for both human medical and veterinary healthcare, i.e. One Health knowledge. For this study, the SPARQL SELECT queries q1V to q3V presented in Step 6 intend to retrieve reliable knowledge for human medicine (UMLS CUI pairs validated with BMJ Best Practice content) about the diagnosis and management of well-known medical conditions that affect humans and animals. We created the SPARQL SELECT queries q1VU to q3VU (see the Additional file 4 for details) that combine the results of the queries q1V to q3V over each dataset VetCN and PMSB. Hence, we report the number of UMLS Metathesaurus concepts pairs with up-to-date clinically meaningful associations for human medicine, although the source data can be from veterinary medicine (i.e. the SAVSNET veterinary clinical narratives) or from human medical science (i.e. the PubMed Systematic Reviews).

The results of the queries q1VU to q3VU as well as the results of the queries q1V to q3V are quantitative. Hence, we can quantify to what extent One Health can provide added value when compared with a conventional approach that will keep both datasets VetCN and PMSB separated for being part of either veterinary medicine or medical science.

A UMLS Metathesaurus concept can be mapped to none, one or more than one SNOMED CT concepts. We created the SPARQL SELECT query q1VM to q3VM (see the Additional file 4 for details) to retrieve from the OBANmod those UMLS CUI pairs validated with BMJ Best Practice content, where the candidate concept is mapped to at least one SNOMED CT concept. Using the OBANmod and the asserted information within each locality-based module as the default graph, we created the SPARQL SELECT query q1VS to q1VS (see the Additional file 4 for details) that retrieves those SNOMED CT concept pairs (OWL Classes) mapped to UMLS CUI pairs validated with BMJ Best Practice content. For these SNOMED CT pairs, it is possible to go beyond the knowledge captured in the OBAN “sometimes true” association relationships.

Each locality-based module created for a well-known medical condition contains asserted as well as inferred knowledge that can expand/enrich the results from the queries q1VS to q3VS by exploiting the transitive closure of rdfs:subClassOf for the SNOMED CT concepts in OWL. The SPARQL SELECT queries q1VR to q3VR (see the Additional file 4 for details) use as the default graph the inferred model obtained with the DL reasoner FaCT++ for each locality-based module. The queries q1VR to q3VR retrieve the OWL Classes that are asserted and inferred descendants of the those SNOMED CT concepts that are mapped to candidate concepts of the SNOMED CT pairs retrieved from the SPARQL SELECT queries q1VS to q3VS.

## Results

We start by illustrating and reporting the results obtained for each step of the SemDeep pipeline using the PMSB and VetCN datasets. Next, we combine the results from the SemDeep pipeline to investigate to what extent the One Health approach can provide added value.

### A SemDeep pipeline

#### Step 1: computing n-grams from unstructured text

As in our previous work [44–47], we employ word2phrase to obtain n-grams and we preserve numbers and punctuation marks including parenthesis as they appear in the unstructured text. However, the original text of the PMSB dataset is not converted to lower case as in [44–47]. The PMSB dataset contains 447 M terms (words and tokens as they appear in the text), and after obtaining the n-grams, this number reduces significantly to 46 M. This means that a high number of tokens/words appear to be repeatedly collocated.

The original text of the VetCN dataset was converted to lower case before computing n-grams as many examples were found within the unstructured text of indiscriminate alternation between lowercase and uppercase. The VetCN dataset contains 149 M terms (words and tokens as they appear in the text), and after obtaining the n-grams, this reduces to 103 M. A plausible reason for the modest numeric reduction is presence of spelling variations and/or errors for the same term. To confirm this hypothesis, we utilise a probabilistic spelling corrector [89] that provides alternative spellings for a word or token appearing within a corpus; the hypothesis was confirmed. For example, the term “vomiting” appears in VetCN with more than 50 spelling variations such as “vomiteting” or “vomittimng” or “vomirtting” or “vomikting” (to mention just a few), which can be considered spelling errors. Furthermore, by close inspection of the unstructured text, it can be observed that short forms like “v” are used instead of the long form “vomiting”.

#### Step 2: creation of neural embeddings

We use the word2vec implementations for CBOW and Skip-gram and apply the same hyperparameter configuration as our previous study [55]. To compute the neural embeddings, we use a Dell PowerEdge R430 with 100GB RAM and 32 virtual CPUs Intel Xeon E5–2690 v4 at 2.6 GHz. With this, creating the neural embeddings with PMSB takes 17 min for Skip-gram and 2 min for CBOW; for VetCN, creating the neural embeddings takes 29 min for Skip-gram and 2 min for CBOW.

For both PMSB and VetCN, we obtained a list of terms (n-grams) with a frequency count greater than 5 and with vector representations of real numbers. For PMSB, 423 K terms have vector representations; for VetCN, 488 K terms have vector representations.

#### Step 3: obtaining term pairs for the semantic similarity and relatedness task

Table 2 shows the target terms that are the subject for this study, i.e. 11 n-grams with the same UMLS CUI corresponding to the medical conditions – UMLS Semantic Type “T047|Disease or Syndrome” – that appear in both PMSB and VetCN with a frequency count greater than 1 K. For each target term in Table 2, it was feasible to find a document in BMJ Best Practice (last column in Table 2) about the medical condition.

**Table 2:** *The target terms for PMSB and VetCN datasets*

Target terms for this study and their concept identifiers in UMLS and SNOMED CT				BMJ Best Practice document
UMLS CUI	SNOMED CT identifier	VetCN dataset n-gram (frequency count)	PMSB dataset n-gram (frequency count)	
C0018801	84,114,007	heart_failure (1292)	heart_failure (4615)	Chronic congestive heart failure
C0004096	195,967,001	asthma (1194)	asthma (8891)	Asthma in adults
C0014544	84,757,009	epilepsy (1164)	epilepsy (3521)	Generalised seizure
C0017601	23,986,001	glaucoma (1657)	glaucoma (1635)	Open-angle glaucoma
C1561643	709,044,004	ckd (2698)	CKD (1550)	Chronic kidney disease
C0029408	396,275,006	osteoarthritis (1765)	osteoarthritis (1991)	Osteoarthritis
C0002871	271,737,000	anaemia (1414)	anaemia (1154)	Assessment of anaemia
C0003864	3,723,001	arthritis (8276)	arthritis (1023)	Rheumatoid arthritis
C0011849	73,211,009	diabetes (3660)	diabetes (12846)	Type 2 diabetes in adults
C0020538	38,341,003	hypertension (1132)	hypertension (8365)	Essential hypertension
C0028754	414,916,001	obesity (1763)	obesity (10030)	Obesity in adults

The last column contains the names and references of BMJ Best Practice documents used for validation in Step 5 (see details within the section Materials and methods). The first column contains the UMLS CUI mapped to a target term (n-gram) with the aid of MetaMap. The second column shows the SNOMED CT identifier mapped to the UMLS CUI with the aid of the UMLS API. The third column displays the target terms from the VetCN dataset, i.e. the n-grams with their frequency counts in the corpus appear within brackets. The fourth column shows the target terms from PMSB dataset with the same format of the third column. All target terms (i.e. n-grams) are identical for both datasets except one. The well-known medical condition “chronic kidney disease” with UMLS CUI = “C1561643” has the n-gram “CKD” (i.e. a short form with all the characters in upper case) in the PMSB dataset; while in VetCN dataset it has the n-gram “ckd”. The difference in these two target terms “CKD” and “ckd” happens as in Step 1, VetCN corpus is transformed to lower case while PMSB corpus is not

We limit the list of the candidate terms (n-grams) to the 20 top-ranked terms. As there are 11 chosen medical conditions (i.e. target terms), the Additional file 1 contains 880 term pairs (target term, candidate term): the worksheet “VetCN” with the 440 term pairs using CBOW and Skip-gram with VetCN; and the worksheet “PMSB” with the 440 term pairs using CBOW and Skip-gram with PMSB.

By visual inspection of the Additional file 1, we observe that the cosine values are systematically higher for the candidate terms obtained from the neural embeddings created with Skip-gram than the cosine values for CBOW. Hence, CBOW learns the vectors quicker than Skip-gram (as reported in Step2), although the vectors obtained for the 20 top-ranked candidate terms are less similar to the target term (lower cosine value) for CBOW than for Skip-gram.

#### Step 4: named entity recognition (NER) task

The NER task can be interpreted as a grounding or normalisation to unveil the semantic meaning of the n-grams, and thus, provide more detail on the extent of semantic overlap between the candidate terms obtained from both datasets. Considering the guidelines introduced in Step 4 (section Materials and methods), UMLS CUIs are assigned to the n-grams to represent the focus concepts corresponding to all 613 unique candidate terms for the 880 term pairs.

Table 3 shows only 20 n-grams that are the only common candidate terms among the 880 term pairs. From Table 3, there is at least one candidate term for all the 11 well-known medical conditions (target terms) that is common to both datasets.

**Table 3:**
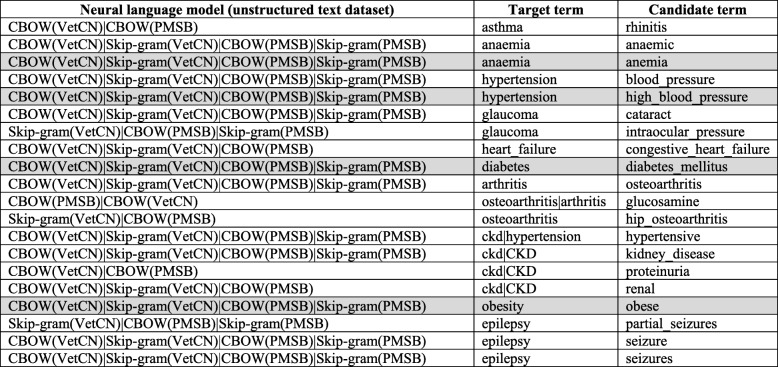
*The 20 n-grams that are the only common candidate terms among the 880 term pairs from both VetCN and PMSB datasets*

The character ‘|’ that appears in the first column separates the different neural language models. The grey background indicates that the target term and the candidate term has the same focus concept, i.e. same UMLS CUI

The columns I, J, and K from both worksheets “VetCN” and “PMSB” from the Additional file 1 have values if the candidate term (n-gram) contains a short form correctly or incorrectly identified by the short form detector. The column I will have the values {SF, SF-U, SF-NU} if a clinically meaningful short form has been correctly identified and the values {SF-I, SF-NF} if the short form detector has made an error. For example, “US” is a short form for the long form “United States”, which per se is not clinically meaningful. Typical examples of the “SF-NF “error are short forms with four characters or more appearing in n-grams from VetCN. For example, “echo” is the abbreviation (i.e. short form) for the long form “echocardiography”. Table 4 has the micro-averaging precision, recall, and F measure for the short form detector.

**Table 4:** *Performance of the short form detector with VetCN and PMSB datasets*

Data set	Unique candidate terms (n-grams)	SF-U + SF-NU + SF	SF-I	SF-NF	n-grams with no clinically meaningful short forms	Detect n-grams with one or more clinically meaningful short forms			Detect n-grams with no clinically meaningful short forms		
						P	R	F	P	R	F
VetCN	300	57	1	14	228	98.28	80.28	88.37	94.21	99.56	96.82
PMSB	333	75	2	0	256	97.40	100	98.68	100	99.22	99.61
						97.78	90.41	93.95	97.07	99.36	98.20

To assess the capability of the short form detector to identify candidate terms (n-grams) with one or more clinically meaningful short forms: the value of the column “SF-U + SF-NU + SF” is interpreted as TP; the value of the column “SF-I” is interpreted as FP; and the value of the column “SF-NF” is interpreted as FN. To assess the capability of the short form detector to identify candidate terms (n-grams) with no clinically meaningful short forms: the value of the column “n-grams with no clinically meaningful short forms” is interpreted as TP; the value of the column “SF-I” is interpreted as FN; and the value of the column “SF-NF” is interpreted as FP. The last row shows the micro-averaging values taking into account the total of 613 unique candidate terms (n-grams) for the 880 term pairs. Abbreviations: P = precision; R = recall; and F = F measure

Tables 5 and 6 show the performance of MetaMap in experiments 1 and 2 for the candidate terms (n-grams) obtained for each target term from the neural embeddings created with CBOW and Skip-gram. To retrace the MetaMap output, the domain experts’ assessment, and the performance calculations, the Additional file 1 contains:

- The worksheet “SF to LF” – it has the 63 long forms for 80 short forms (including variants of the short forms) within the candidate terms (n-grams) for the VetCN and PMSB datasets.
- Within both worksheets “VetCN” and “PMSB” – the column G has the UMLS CUIs for the focus concepts of the candidate terms (n-grams). The labels (i.e. {SM, MM, IM, NM}) assigned in EXP-1 by the domain experts to MetaMap output appear within the column H and the labels assigned in EXP-2 by the domain experts to MetaMap output appear in column L.
- The worksheet “MetaMap performance” – it contains the number of TP, FP, and FN obtained and used to calculate precision, recall, and F measure for MetaMap in EXP-1 and EXP-2.

**Table 5:** *MetaMap performance for the candidate terms from VetCN dataset*

Target term (n-gram)	Candidate terms (20 top-ranked n-grams) from CBOW neural embeddings for a target term						Candidate terms (20 top-ranked n-grams) from Skip-gram neural embeddings for a target term					
	MetaMap Experiment 1			MetaMap Experiment 2			MetaMap Experiment 1			MetaMap Experiment 2		
	P	R	F	P	R	F	P	R	F	P	R	F
anaemia	84.21	94.12	88.89	95.00	100.00	97.44	94.74	94.74	94.74	95.00	100.00	97.44
arthritis	93.33	73.68	82.35	100.00	75.00	85.71	94.44	89.47	91.89	100.00	90.00	94.74
asthma	100.00	90.00	94.74	100.00	95.00	97.44	89.47	94.44	91.89	100.00	100.00	100.00
ckd	68.75	73.33	70.97	100.00	95.00	97.44	62.50	71.43	66.67	100.00	100.00	100.00
diabetes	76.47	81.25	78.79	100.00	90.00	94.74	88.89	88.89	88.89	94.74	94.74	94.74
epilepsy	100.00	90.00	94.74	100.00	95.00	97.44	100.00	90.00	94.74	100.00	95.00	97.44
glaucoma	87.50	77.78	82.35	94.74	94.74	94.74	93.33	73.68	82.35	94.74	94.74	94.74
heart_failure	73.68	93.33	82.35	95.00	100.00	97.44	84.21	94.12	88.89	100.00	100.00	100.00
hypertension	71.43	62.50	66.67	100.00	95.00	97.44	72.22	86.67	78.79	100.00	100.00	100.00
obesity	75.00	100.00	85.71	85.00	100.00	91.89	84.21	94.12	88.89	89.47	94.44	91.89
osteoarthritis	94.74	94.74	94.74	100.00	95.00	97.44	85.00	100.00	91.89	85.00	100.00	91.89
	84.10	84.61	83.85	97.25	94.07	95.38	86.27	88.87	87.24	96.27	97.17	96.63

The table shows the performance of MetaMap in Experiment 1 (applying MetaMap to the candidate terms) and Experiment 2 (short form detection and expansion into long form before applying MetaMap to the candidate terms) for each target term (n-gram for a well-known medical condition). The candidate terms are a list of the 20 top-ranked terms (highest cosine value) obtained from the created neural embeddings with CBOW or Skip-gram taking the vector for a target term. The last row shows the average of each evaluation measure over all 11 medical conditions under study to get an overall measure of performance (a.k.a. macro-averaging). Abbreviations: P = precision; R = recall; and F = F measure

**Table 6:** *MetaMap performance for the candidate terms from PMSB dataset*

Target term (n-gram)	Candidate terms (20 top-ranked n-grams) from CBOW neural embeddings for a target term						Candidate terms (20 top-ranked n-grams) from Skip-gram neural embeddings for a target term					
	MetaMap Experiment 1			MetaMap Experiment 2			MetaMap Experiment 1			MetaMap Experiment 2		
	P	R	F	P	R	F	P	R	F	P	R	F
anaemia	90.00	100.00	94.74	85.00	100.00	91.89	84.21	94.12	88.89	94.74	94.74	94.74
arthritis	88.89	88.89	88.89	89.47	94.44	91.89	100.00	100.00	100.00	95.00	100.00	97.44
asthma	76.47	81.25	78.79	72.22	86.67	78.79	63.16	92.31	75.00	68.42	92.86	78.79
CKD	100.00	100.00	100.00	100.00	100.00	100.00	90.00	100.00	94.74	90.00	100.00	94.74
diabetes	63.16	92.31	75.00	68.42	92.86	78.79	75.00	100.00	85.71	80.00	100.00	88.89
epilepsy	85.00	100.00	91.89	95.00	100.00	97.44	90.00	100.00	94.74	95.00	100.00	97.44
glaucoma	90.00	100.00	94.74	100.00	100.00	100.00	84.21	94.12	88.89	100.00	100.00	100.00
heart_failure	85.00	100.00	91.89	90.00	100.00	94.74	73.68	93.33	82.35	90.00	100.00	94.74
hypertension	95.00	100.00	97.44	100.00	100.00	100.00	84.21	94.12	88.89	95.00	100.00	97.44
obesity	100.00	95.00	97.44	100.00	100.00	100.00	94.74	94.74	94.74	95.00	100.00	97.44
osteoarthritis	90.00	100.00	94.74	100.00	100.00	100.00	90.00	100.00	94.74	100.00	100.00	100.00
	87.59	96.13	91.41	90.92	97.63	93.96	84.47	96.61	89.88	91.20	98.87	94.70

The table shows the performance of MetaMap in Experiment 1 (applying MetaMap to the candidate terms) and Experiment 2 (short form detection and expansion into long form before applying MetaMap to the candidate terms) for each target term (n-gram for a well-known medical condition). The candidate terms are a list of the 20 top-ranked terms (highest cosine value) obtained from the created neural embeddings with CBOW or Skip-gram taking the vector for a target term. The last row shows the average of each evaluation measure over all 11 medical conditions under study to get an overall measure of performance (a.k.a. macro-averaging). Abbreviations: P = precision; R = recall; and F = F measure

The last row of Tables 5 and 6 shows the macro-averaging precision, recall and F measure. Taking the 613 unique candidate terms for the 880 term pairs, the micro-averaging precision of MetaMap is 84.68% for EXP-1 and 92.41% for EXP-2; the micro-averaging recall of MetaMap is 91.44% for EXP-1 and 96.48% for EXP-2; hence, the micro-averaging F measure of MetaMap is 87.93% in EXP-1 and 94.40% in EXP-2.

The macro-averaging and the micro-averaging values for precision, recall, and F measure are consistently higher in EXP-2 than in EXP-1. However, when examining the performance of MetaMap in Tables 5 and 6, two exceptions are noticeable (two rows in Table 6 with a grey background) where a drop in performance for EXP-2 can be observed:

1. The target term “anaemia” has precision and F measure higher in EXP-1 than in EXP-2. Both n-grams “g/dL” and “g/dl” can be mapped by MetaMap in EXP-1 to the UMLS Metathesaurus concept “C0439267|Gram per Deciliter”, which is more specific than the UMLS Metathesaurus concept “C1519795|Unit of Measure”. The UMLS CUI = C1519795 is automatically assigned by the short form detector in EXP-2.
2. The target term “arthritis” has precision and F measure higher in EXP-1 than in EXP-2. The n-gram “Cox-2_inhibitors” is mapped by MetaMap in EXP-1 to the UMLS Metathesaurus concept “C1257954|Cyclooxygenase 2 Inhibitors”. As “Cox-2” is the short form for the long form “cycloxygenase-2”, the n-gram “Cox-2_inhibitors” was expanded into “cycloxygenase-2_inhibitors” for EXP-2. However, MetaMap in EXP-2 did not map the expanded n-gram to the UMLS CUI = C1257954.

Following Smucker et al. [69], we apply the Student’s paired t-test [70] taking the values from Tables 5 and 6 for EXP-1 and EXP-2 to measure the statistical significance of the difference in the mean average precision, recall, and F measure. We obtain the following *p*-values: 0.0001046 for precision, 0.001077 for recall, and 0.00001191 for F measure. As the p-values obtained with the Student’s paired t-test are significantly small, we reject that “*the difference in averages could be due to chance*” [67].

The outcome of this step is a set of 342 unique UMLS Metathesaurus concept pairs (target concept, candidate concept) that represent the focus concept pairs for the 880 n-gram pairs (target term, candidate term). The next step will determine if there is a clinically meaningful association for each UMLS Metathesaurus concept pair.

#### Step 5: validation of the term pairs mapped to UMLS Metathesaurus concept pairs using BMJ best practice

The Additional file 3 contains the 3-tuples (target concept, candidate concept, validation label) for the VetCN dataset (worksheet “VetCN”) and the PMSB dataset (worksheet “PMSB”). Among the 35 unique UMLS Metathesaurus concept pairs that are common to both datasets, there are 10 concept pairs with the validation label “`Itself”, i.e. one per chosen medical condition (target term) with the only exception being “chronic kidney disease” (i.e. ckd). Table 7 displays the 25 unique UMLS Metathesaurus concept pairs common to both datasets and with validation labels different than “itself”.

**Table 7:**
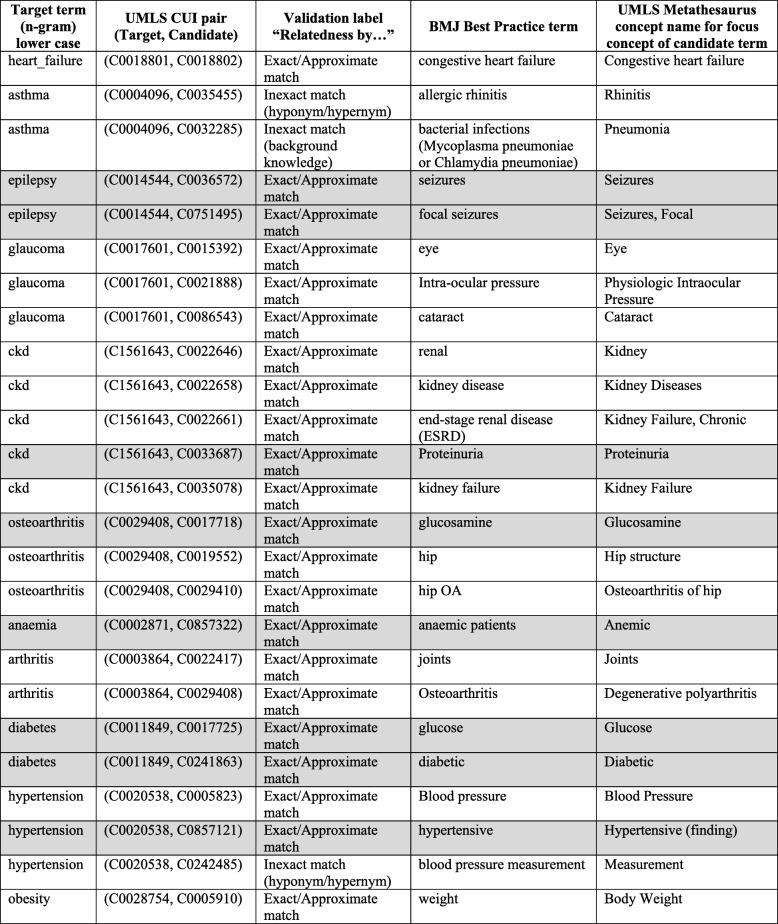
The 25 unique UMLS Metathesaurus concept pairs that are common to both VetCN and PMSB datasets and have validation labels different to “itself”

The validation label from the third column denotes the relatedness of BMJ Best Practice term (the column previous to the last) to the UMLS Metathesaurus concept representing the focus concept for the candidate term (the last column). The rows with grey background correspond to UMLS Metathesaurus concept pairs that are retrieved by the SPARQL queries q1VU, or q2VU, or q3VU (see the cells with grey background in Table 11 for details)

The last column of Table 7 shows the UMLS Metathesaurus concept name representing the focus concepts for the candidate term. This column does not appear in the Additional file 3 as this information can be obtained with the UMLS API. The UMLS API can typically retrieve alternative names for a UMLS Metathesaurus concept in different terminologies, and thus, the Additional file 3 just provides the terminologies’ identifiers (e.g. MeSH: D000068256) instead of the textual excerpts from terminological resources.

Table 8 displays the 11 UMLS Metathesaurus concept pairs that were identified by the domain experts as unrelated as they do not have an up-to-date clinically meaningful association. These 11 UMLS Metathesaurus concept pairs correspond to only five of the total of 11 medical conditions (i.e. target term) that are the subject of this study. For eight UMLS Metathesaurus concept pairs, the candidate concept could not be interpreted per se as clinically meaningful in connection with the medical condition, and thus, they have assigned the label “Unrelated: not clinically meaningful”. For three UMLS Metathesaurus concept pairs, which corresponds to the medical condition epilepsy and glaucoma, the validation label “Unrelated: excluded” was assigned and the rationale behind is the following:

- UMLS Metathesaurus concept pair (C0014544|Epilepsy, C0071754|potassium bromide): in medicine “potassium bromide” is no longer a suitable treatment for epilepsy due to side effects [90] (see correspond excerpt in evalBMJ.xls).
- UMLS Metathesaurus concept pair (C0014544|Epilepsy, C3886490|Pexion): the active substance of pexion is imepitoin, which is a type of partial benzodiazepine site agonists. In medicine “benzodiazepine” is no longer a suitable treatment for epilepsy due to adverse effects [91] (see corresponding excerpt in evalBMJ.xls).
- UMLS Metathesaurus concept pair (C0017601|Glaucoma, C0014392|Enucleation procedure): As “malignant melanomas of the ciliary body” does not appear either explicitly or implicitly in BMJ Best Practice for open-angle glaucoma [92], which is the medical condition for which the “enucleation procedure” is recommended [93], the concept pair is judged as unrelated. As “enucleation of glaucomatous eyes” does appear in veterinary literature, another excerpt was added to capture this fact. For further details, see corresponding excerpts in evalBMJ.xls.

**Table 8:**
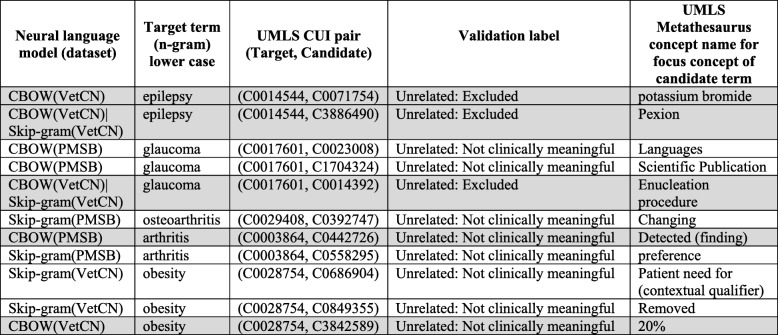
The 11 UMLS Metathesaurus concept pairs identified by the domain experts as unrelated, as they do not have an up-to-date clinically meaningful association for human medicine.

The rows with grey background correspond to UMLS Metathesaurus concept pairs that are retrieved for some of the SPARQL queries q1 to q3 (see the cells with grey background in Table 9 for details)

As the above-mentioned three UMLS Metathesaurus concept pairs are up-to-date clinically meaningful associations in veterinary healthcare, they denote differences between human and veterinary healthcare.

The Additional file 3 contains a total of 342 unique 3-tuples considering both the VetCN and PMSB datasets, of which only 11 have UMLS Metathesaurus concept pairs that are considered as unrelated. The UMLS Metathesaurus concept pairs in Table 8 are considered False Positives (FP), and likewise the term pairs that have them as focus concepts. All other UMLS Metathesaurus concept pairs not included in Table 8 are considered as True Positives (TP), and likewise the term pairs that have them as focus concepts. Hence, it is feasible to calculate precision and estimate the performance of CBOW and Skip-gram for the semantic similarity and relatedness task using the 880 term pairs:

- For VetCN, precision is 98.18% for both CBOW and Skip-gram.
- For PMSB, the precision is 98.64% for CBOW and 99.09% for Skip-gram.

The BMJ health informatician considered it worthy to report to BMJ Best Practice editors that:

1. Some concept pairs do not have candidate concepts that are easily found within BMJ Best Practice for the medical condition. Hence, external evidence from systematic research was needed to validate them, and thus, the concept pairs have assigned the validation label “Relatedness by Inexact match (background knowledge)” instead of the expected label “Relatedness by exact/approximate match”. For example, for the concept pair (C0004096|Asthma, C0006277|Bronchitis), bronchitis is not mentioned as a differential diagnosis in BMJ Best Practice for asthma, although BMJ Best Practice for acute bronchitis has asthma as a differential. Another example is the concept pair (C0004096|Asthma, C0232602|Retching), where retching does not currently appear along with coughing in the description of symptoms within BMJ Best Practice for asthma.
2. Some concept pairs have candidate concepts that may enhance the current content of BMJ Best Practice for the medical condition. For example, for the concept pair (C0017601|Glaucoma, C0020581|Hyphema), where hyphema appears as a common postoperative complication in patients with glaucoma that had trabeculotomy (a.k.a. glaucoma filter surgery). This concept pair has the validation label “Relatedness by Inexact match (background knowledge)” and can be interpreted as an example of transitivity: glaucoma has trabeculotomy as a surgical procedure, and trabeculotomy has hyphema as a common complication. Only by making “trabeculotomy” explicit does the relatedness for the concept pair (glaucoma, hyphema) become transparent. It should be noted that the relationship between “trabeculotomy” from BMJ and “C0020581|Hyphema” is a cause-effect relationship.

#### Step 6: formal representation of the knowledge acquired and validated

The third column of Table 1 shows OWL individuals for the main OWL Classes of the lemonEXT and OBANmod created programmatically applying the axiom patterns from the second column in Table 1. We use the reasoner FaCT++ to check that – once populated – the lemonEXT and OBANmod ontologies are consistent (i.e. no unsatisfiable classes).

Once the lemonEXT core ontology is populated with the term pairs (target term, candidate term) from VetCN, including their focus concepts from the UMLS Metathesaurus, it contains a total of 3477 axioms (class count: 324; individual count: 502) and its DL expressivity is ALEI. Once the lemonEXT is populated with the term pairs from PMSB, including their focus concepts, it contains a total of 3702 axioms (class count: 338; individual count: 549) and its DL expressivity is ALEI.

Once the OBANmod core ontology is populated with the 192 unique 3-tuples from VetCN, it contains a total of 5893 axioms (class count: 347; individual count: 842) and its DL expressivity is ALEHI(D). Once the OBANmod is populated with the 185 unique 3-tuples from PMSB, it contains a total of 5545 axioms (class count: 361; individual count: 781) and its DL expressivity is ALEHI(D).

**Fig. 4:**
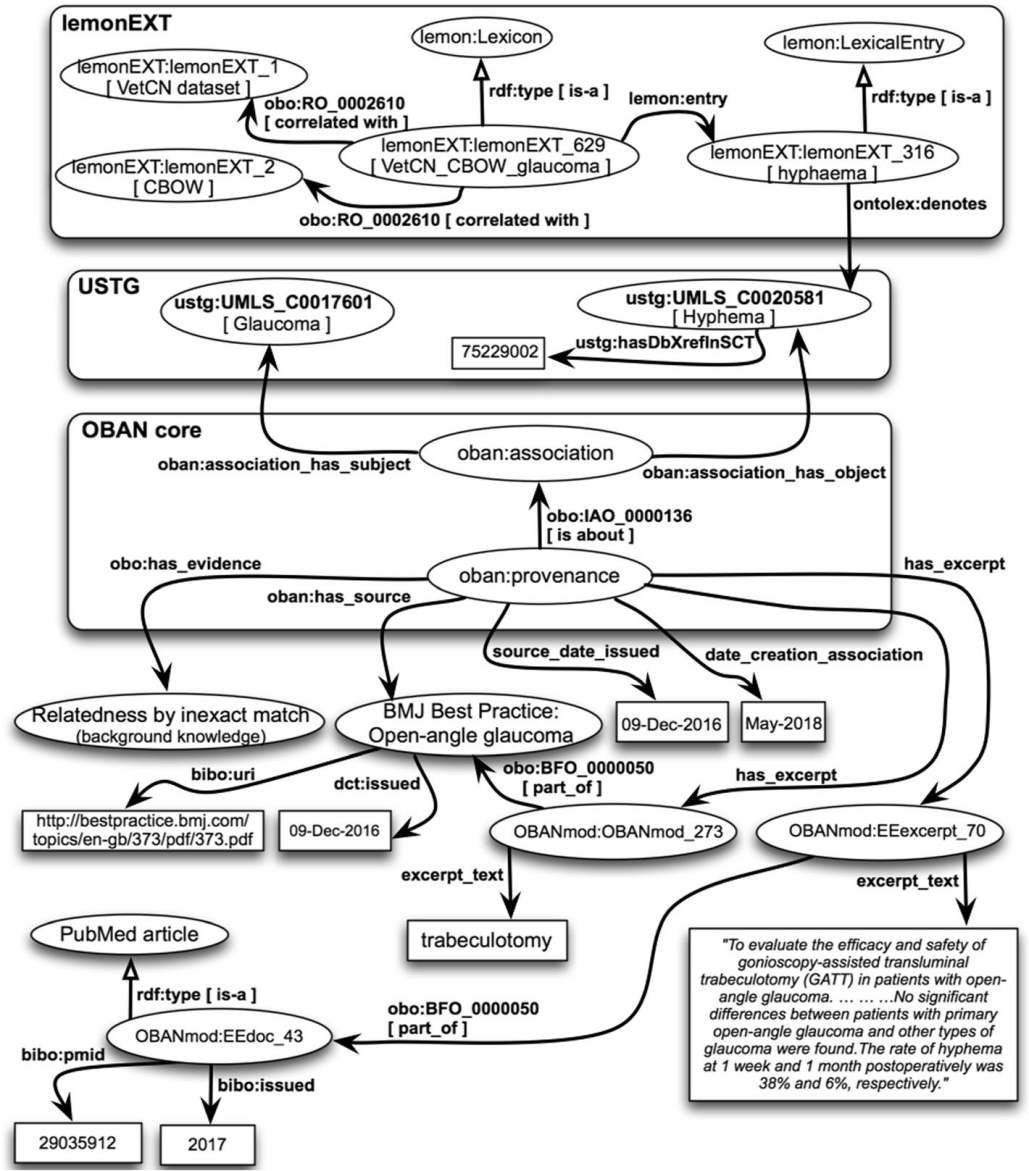
*Exemplifying the population of both the lemonEXT and OBANmod core ontologies* – the figure illustrates how the 3-tuple (C0017601|Glaucoma, C0020581|Hyphema, “Relatedness by Inexact match (background knowledge)”) is represented as a modified version of the OBAN association. This 3-tuple has two bibo:excerpts: 1) the term “trabeculotomy” from BMJ Best Practice for open-angle glaucoma; and 2) few lines of text from the PubMed article with identifier PMID = 29,035,912. The top of the figure shows the lexical entry from the lemonEXT ontology corresponding to the focus concept C0020581|Hyphema

To illustrate the population of both the lemonEXT and OBANmod core ontologies, Fig. 4 takes as an example the term pair (glaucoma, hyphaema) from the neural embeddings created with CBOW using VetCN dataset. This term pair corresponds to the 3-tuple (C0017601|Glaucoma, C0020581|Hyphema, “Relatedness by Inexact match (background knowledge)”), and Fig. 4 displays how this 3-tuple is represented as a “sometimes true” association relationship in OWL with evidence-based provenance. The top of Fig. 4 shows the candidate term (i.e. an n-gram represented as a lexical entry from the lemonEXT) with a UMLS Metathesaurus concept as the focus concept with CUI = C0020581, which corresponds to the SNOMED CT concept with identifier = 75,229,002.

Table 9 contains the number of UMLS Metathesaurus concept pairs retrieved from executing: a) the SPARQL SELECT queries q1 to q3 over the lemonEXT populated for each dataset VetCN and PMSB separately; and b) the SPARQL SELECT queries q1V to q3V over the OBANmod populated for each dataset VetCN and PMSB separately.

**Table 9:**
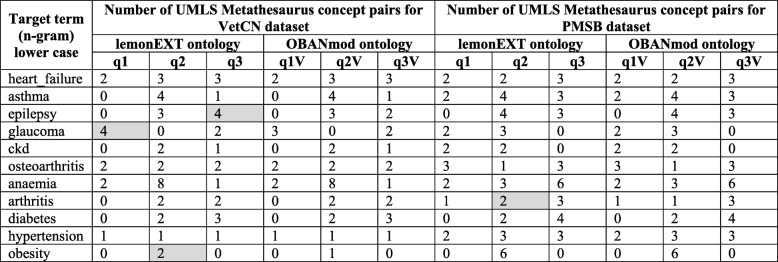
*Results of the SPARQL SELECT queries performed over the lemonEXT ontology and the OBANmod ontology* – The table shows the number of UMLS Metathesaurus concept pairs (target, candidate) retrieved for the SPARQL SELECT queries q1 to q3 as well as q1V to q3V

The SPARQL SELECT queries appear within the Additional file 4 and the description of the queries appear within the Step 6 of the section Materials and methods. Each UMLS Metathesaurus concept pair represents the focus concepts of the term pair (target term, candidate term). The difference in the number of results between the query qi and the query qiV, with i = {1,2,3}, indicates that there are UMLS Metathesaurus concept pairs that have not passed the evaluation with BMJ Best Practice, and thus, the query qi (see cells with grey background) has a higher number of results than the query qiV

### Extracting locality-based modules with SNOMED CT and enabling one health queries

The worksheet “signatures” within the Additional file 3 contains the ontological signature (i.e. list of SNOMED CT identifiers) for each of the 11 medical conditions that are the subject of this study. We use the ModuleType BOT from the OWL API to create upper modules (a.k.a. locality-based modules). With a MacBook Pro Retina with 2.8 GHz Intel Core i7 and 16GB of RAM memory, the average time to create a locality-based module is 8 h.

All the locality-based modules created have a DL expressivity ALER, i.e. the same as the SNOMED CT ontology. Table 10 shows the details for each locality-based module extracted. From Table 10 it is easy to observe many axioms for the locality-based module extracted for diabetes mellitus. Although the guidelines introduced in Step 4 intend to avoid selection of focus concepts for the candidate terms that are too general for the medical condition (see the Additional file 2 for details), due to gaps in the terminological resources within the UMLS Metathesaurus, it may be that the focus concepts for some candidate terms (n-grams) are too broad in the biomedical or clinical meaning. Indeed, this is the case for diabetes mellitus. The UMLS Metathesaurus concept pair (C0011849|Diabetes Mellitus, C0012634|Disease) has the focus concepts for the term pair (“diabetes”, “concurrent_diseases”), which was obtained with Skip-gram using VetCN. As the UMLS Metathesaurus concept “C0012634|Disease” was selected as the focus concept for the candidate term (n-gram) “concurrent_diseases”, the SNOMED CT concept “64572001| Disease (disorder)” that maps the UMLS CUI = C0012634 is included in the ontological signature for diabetes mellitus. It should be noted that in the January 2017 version of SNOMED CT in OWL, the OWL Class for “64,572,001| Disease (disorder)” has 1810 OWL Classes as asserted descendants and 72,253 OWL Classes as asserted as well as inferred descendants using the reasoner FaCT++. Furthermore, one or more attribute-value pairs in an asserted or inferred descendant will trigger extraction of all axioms contributing the descendant’s meaning.

**Table 10:** Locality-based modules extracted from the SNOMED CT ontology for the 11 well-known medical conditions

Target term (n-gram) lower case	Number of SNOMED CT concept identifiers for the signature	Total number of axioms	Number of OWL Classes	Number of OWL object properties	Number of SubClassOf axioms	Number of EquivalentClass axioms
anaemia	34	105,205	22,227	46	7092	15,134
arthritis	33	74,398	16,180	31	7470	8709
asthma	37	97,647	20,804	39	7611	13,192
ckd	19	51,890	10,929	40	3899	7029
diabetes	29	463,437	100,119	44	49,818	50,300
epilepsy	19	10,072	2085	23	1055	1029
glaucoma	39	101,997	21,278	44	11,047	10,230
heart_failure	37	68,006	14,634	44	5490	9143
hypertension	31	152,283	32,270	48	16,425	15,844
obesity	28	90,224	18,994	40	12,376	6617
osteoarthritis	38	73,496	15,903	37	8922	6980

The second column just reports the total number of SNOMED CT identifiers for the ontological signature. The worksheet “signatures” within the Additional file 3 contains the list of SNOMED CT identifiers (as signature) for each target term. From the third to the last column ontology metrics information for the locality-based module created per target term is provided. The last two columns indicate the number of descriptions and definitions extracted from the SNOMED CT ontology for each locality-based module, respectively

Table 11 contains the results of executing the “One Health” queries proposed for this study. The worksheet “q One Health” within the Additional file 3 contains the number of UMLS CUI pairs validated with BMJ Best Practice content (i.e. human medicine) for each of the 27 UMLS Semantic Types that participates in the SPARQL SELECT query q1VU or q2VU or q3VU.

**Table 11:**
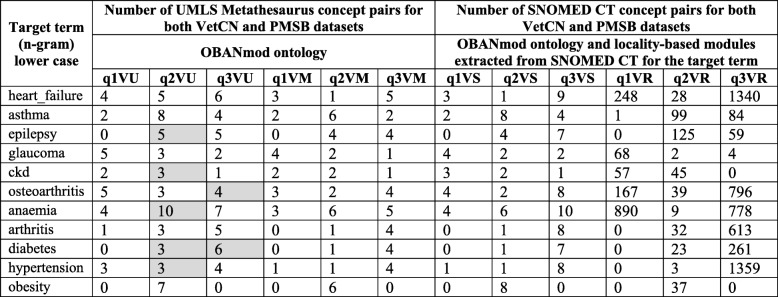
*Results of the “One Health” queries performed that intend to acquire validated knowledge about the diagnosis and management of well-known medical conditions* – The SPARQL SELECT queries appear within the Additional file 4 and the description of the queries appear within the subsection “Extracting locality-based modules with SNOMED CT and enabling One Health queries” of the section Materials and methods

Each query qiVU, with i = {1,2,3}, is the union of the results obtained for the query qiV over VetCN dataset and the query qiV over PMSB dataset (see Table 9 for details). The cells with grey background indicate that there are common UMLS Metathesaurus concept pairs in both VetCN and PMSB datasets, and therefore, the total number of results for the query qiVU is lower that the summation of the results obtained for the query qiV in each dataset (see the rows with grey background in Table 7 for details of the common UMLS Metathesaurus concept pairs retrieved). As more than one SNOMED CT concept can map one UMLS Metathesaurus concept, the number of results for the query qiVM is equal to or lower than the number of results for the query qiVS, with i = {1,2,3}. Each SPARQL SELECT query qiVR, with i = {1,2,3}, retrieves the asserted and inferred descendants (with FaCT++) of those SNOMED CT concepts mapped to candidate concepts of the SNOMED CT pairs retrieved from the SPARQL SELECT query qiVS

According to Table 11:

1. The SPARQL SELECT queries q1VU to q3VU obtain equal or more UMLS CUI pairs validated with BMJ Best Practice content (i.e. human medicine) than the queries q1V to q3V over VetCN and PMSB separately (Table 9). Hence, the higher number of up-to-date clinically meaningful associations for human medicine demonstrates that a “One Health” approach can provide added value by combining the UMLS CUI pairs (number of associations) from veterinary medicine (i.e. the SAVSNET veterinary clinical narratives) and from human medical science (i.e. the PubMed Systematic Reviews). The cells with a grey background indicate the existence of common UMLS CUI pairs in both datasets VetCN and PMSB.
2. The query q2VU obtains UMLS CUI pairs validated with BMJ Best Practice content (i.e. human medicine) for all 11 target terms (i.e. the chosen medical conditions). By looking at worksheet “q One Health” within the Additional file 3 and examining the number of UMLS CUI pairs obtained for the UMLS Semantic Type “T033|Finding” alone, we can safely state that our SemDeep approach has captured “sometimes true” association relationships between each well-known medical condition and its clinical findings. Hence, “One Health” knowledge useful for the diagnosis of each of the 11 well-known medical conditions has been obtained.
3. By combining the results of the queries q1VU and q3VU, it may be possible to obtain the UMLS CUI pairs validated with BMJ Best Practice content (i.e. human medicine) for the management of each well-known medical condition. We looked at worksheet “q One Health” within the Additional file 3 and examined the number of UMLS CUI pairs obtained for: a) the UMLS Semantic Type “T061|Therapeutic or Preventive Procedure” (included in query q1VU); and b) the UMLS Semantic Type “T121|Pharmacologic Substance”, which belongs to the UMLS Semantic Group CHEM (included in query q3VU). Taking into account the number of UMLS CUI pairs for both UMLS Semantic Types, we can safely state that our SemDeep approach has captured “sometimes true” association relationships useful for the management of each medical condition with the only exception of obesity. According to BMJ Best Practice for obesity in adults [94], the first line of treatment for obesity is diet. There are candidate terms (n-grams) from VetCN dataset that have as focus concept the UMLS Metathesaurus “C0012155|Diet”, which has the UMLS Semantic Type “T168|Food”, and therefore, outside of the 27 UMLS Semantic Types involved in the SPARQL SELECT queries q1VU to q3VU. For some n-grams containing the token “diet” MetaMap provided also the UMLS Metathesaurus concept “C0012159|Diet (Diet therapy) “that has the UMLS Semantic Type “T058|Health Care Activity”, and thus, within the scope of query q1VU. The UMLS Metathesaurus concept “C0419178|Dietary regime”, which has the UMLS Semantic Type “T061|Therapeutic or Preventive Procedure “was not provided by MetaMap for any n-gram with the token “diet” or “diets”. Taking into account the two UMLS CUIs provided by MetaMap, the UMLS CUI = C0012155 was chosen instead of the UMLS CUI = C0012159 following the guidelines introduced in Step 4 of Materials and Method as the UMLS CUI = C0012155 is broader in meaning and it is mapped to SNOMED CT. Hence, after a closer inspection, as the UMLS CUI pair (C0028754|Obesity, C0012155|Diet) has passed the validation with the BMJ Best Practice content, we can safely state that our SemDeep approach has captured “sometimes true” association relationships useful for the management of each of the 11 well-known medical conditions including obesity.
4. Looking at the results of the queries q1VS to q1VS and taking into account that the UMLS CUI pair (C0028754|Obesity, C0012155|Diet) is mapped to SNOMED CT, it seems reasonable to conclude that SNOMED CT concepts useful for the diagnosis/management of the 11 medical conditions have been obtained. Hence, SemDeep can acquire “One Health” knowledge from veterinary medicine (VetCN) and medical sciences (PMSB) that can be reused and shared among organisations, i.e. there are sets of SNOMED CT concepts relevant for the diagnosis/management of each of the 11 well-known medical conditions (i.e. diseases or syndromes) under study.
5. Looking at the results of the queries q1VR to q1VR, we can safely state that there are asserted and inferred descendants for some of the SNOMED CT concepts relevant for the diagnosis/management of the 11 diseases that affect humans and animals. Hence, the knowledge within the locality-based modules extracted from the SNOMED CT ontology for each well-known medical condition can be used to enrich/expand the sets of SNOMED CT concepts mapped to the UMLS CUI pairs validated with BMJ Best Practice content (i.e. human medicine).

## Discussion

Semantic Deep Learning can be used to generate embeddings of nodes that contain both implicit and explicit information within biological knowledge graphs [95]. A knowledge graph is a “*graph-based representation of entities in the world and their interrelations*” [95]. In previous work, Jauhar et al. [96] adapted a neural language model (e.g. Skip-gram) to generate ontologically grounded sense vectors by adding latent variables representing senses and assuming a fixed ontology [96]. Other work combines both text corpora and ontologies, for example by requiring that the neural embeddings of two terms are similar if the two terms have a semantic relationship in the ontology [97]; this is complementary to retrofitting vectors (i.e. post-processing) to an ontology [96].

The work here transforms neural embeddings of n-grams into an augmented biomedical dataset of normalised and interlinked UMLS Metathesaurus concepts by reusing existing ontologies (i.e. lemon, OBAN, and SNOMED CT) that provide the foundation to acquire reusable One Health knowledge about well-known diseases in humans and animals. The approach is an external intervention in which neural embeddings are not modified but enhanced.

Associating vector representations to terms is the foundation of both classical distributional representations (e.g. LSA and LDA) and distributed representations (i.e. neural embeddings). Despite differences between these approaches (see [98] for details) a shared common problem is that most vector-space models represent a term with one vector, and thus, they do not capture homonymy and polysemy that require multiple vector representations per term [99]. In this study, assigning one or more focus concepts from UMLS Metathesaurus to neural embeddings of n-grams – where each UMLS Metathesaurus concept can have one or more senses (i.e. UMLS Semantic Types) – can be interpreted as a way to restore the homonymy and polysemy inherent in biomedical/clinical terms.

Inclusion of related terms is critical when querying PubMed or clinical narratives with the aim to help health professionals answer questions about patient care. Pakhomov et al. [25] acknowledge that when querying clinical narratives for a medical condition such as “heart failure” often requires additional terms, such as “pulmonary edema” or “shortness of breath”, i.e. related terms that, without being synonyms to the underlying medical condition, denote different aspects of the same underlying medical condition. Similarly, the use of related terms in PubMed searches – either in the form of text words or MeSH terms – has been acknowledged as key to develop successful literature searches that yield meaningful results [100]. For example, to answer the focused clinical question “*in patients with sepsis (population), does treatment with steroids (intervention) compared with no steroids (comparison) alter mortality (outcome)?*” [101], the related term “septic shock” or “severe sepsis” can be used instead of the term “sepsis”. Indeed, a PubMed search for systematic reviews of “severe sepsis” and “steroids” identified fewer useful articles than the more general search for “sepsis” and “steroids” [101]. It should be noted that a) narrowing a PubMed search to fewer useful articles is critical for retrieving a quantity of articles that is manageable; and b) the Cochrane Handbook for Systematic Reviews of Interventions [102] established PICO (Participants, Interventions, Comparisons and Outcomes) as the basis to prepare and maintain Cochrane systematic reviews, which lay the foundations for evidence-based medicine. PICO is typically applied when formulating focused clinical question as above.

Term pairs like (“heart failure”, “pulmonary edema”) or (“severe sepsis”, “steroids”) do not appear among the main four similarity and relatedness benchmarks [27] that are specific to the medical/clinical domain. Furthermore, in total there are fewer than 1 K term pairs in these benchmarks, whilst the 2018AA Metathesaurus release contains 3.67 M concepts and 14 M unique concept names [103]. McInnes et al. [104] developed the UMLS-Similarity that exploits both hierarchical and non-hierarchical information in the UMLS Metathesaurus (i.e. the MRREL table). UMLS-Similarity has two limitations. On the one hand, if the underlying terminologies in the UMLS Metathesaurus (e.g. SNOMED CT or MeSH) do not have pairwise relations, such as (“heart failure”, “pulmonary edema”), they will not be included in the MRREL table. On the other hand, the semantic similarity measures included within UMLS-Similarity provide at most a numeric value indicating the relatedness of two UMLS CUIs with no further information. For example, using the UMLS-Similarity Web Interface [105] with the CUI pair (C0018801, C0034063) corresponding to the term pair (“heart failure”, “pulmonary edema”), we obtain:

- “*The relatedness of Heart failure, NOS (C0018801) and Pulmonary edema, NOS (C0034063) using Adapted Lesk (lesk) is 4134*”; for further information about the Lesk measure see [106].
- “*The relatedness of Heart failure, NOS (C0018801) and Pulmonary edema, NOS (C0034063) using Vector Measure (vector) is 0.753*”; for further information about vector measure see [106].

This paper uses evidence-based resources as a mechanism to validate the similarity and relatedness of term pairs such as (“heart failure”, “pulmonary edema”) while providing one or more textual excerpts of external evidence from systematic research or terminological resources. The focus concept pair (C0018801|Heart failure, C0034063| Pulmonary Edema) appears in the Additional file 1 within the worksheet “VetCN” and has the validation label “Relatedness by exact/approximate match” as the term “pulmonary oedema” appears within BMJ Best Practice for chronic congestive heart failure [85], and thus, there is current best evidence of “pulmonary edema” being clinically related to “heart failure” (see details in the Background section). An advantage of our proposal is providing evidence-based provenance for the association (semantic relatedness) where the evidence-based source is available for the clinicians to consult.

The validation with content from BMJ Best Practice is limited to only one document per medical condition. For example, for the “asthma” medical condition, BMJ Best Practice for “asthma in adults” [107] was chosen. However, if content of BMJ Best Practice for asthma was more broadly taken, for example including “asthma in children” [108], more 3-tuples (target concept, candidate concept, validation label) would have the validation label “Relatedness by exact/approximate match”. Currently, the validation label “Relatedness by exact/approximate match” appears in: a) 206 of the total of 342 unique 3-tuples; b) 95 of the total 192 unique 3-tuples for VetCN; and c) 133 of the total 185 unique 3-tuples for PMSB. So overall, 60% of the 342 unique 3-tuples are “grounding” or “normalising” terms within the 11 documents of BMJ Best Practice. This percentage increases to 72% for the 3-tuples from the PMSB dataset, and drops to 49% for the 3-tuples from the VetCN dataset. As BMJ Best Practice documents need frequent updates from the literature, it is worth noticing the contribution to normalisation made from the PMSB dataset.

It has been acknowledged that “*MetaMap’s greatest weakness is its reduced accuracy in the presence of ambiguity*” [109]. This is particularly so when short forms appear within the n-grams. This study implements a new short form detector, which deals with n-grams from both biomedical/clinical documents, and has demonstrated how precision, recall, and F measure of MetaMap can be improved if short form detection and expansion into long form is performed before applying MetaMap to n-grams (EXP-2). The difference in MetaMap performance between EXP-1 and EXP-2 is aligned with several studies [35–37] that have shown shortcomings of UMLS when dealing with short forms.

The assessment of MetaMap output has required close collaboration amongst three domain experts due to the large volume of MetaMap output [109]. In this study, MetaMap produced 2627 possible UMLS CUIs for the set of 613 n-grams. Another difficulty is a noticeable lack of mappings from MetaMap, such as:

- The n-gram “(HFpEF)” is a candidate term for the target term “heart_failure” from the neural embeddings created with Skip-gram using PMSB. “(HFpEF)” can be expanded into “(heart failure with preserved ejection fraction)” with Allie. Although the UMLS Metathesaurus concept “C3889077|Heart failure with preserved ejection fraction” exists in the UMLS releases of 2016, MetaMap could not do the mapping.
- The n-gram “macrovascular_disease” is a candidate term for the target term “diabetes” from the neural embeddings created with Skip-gram using PMSB. Although the UMLS Metathesaurus concept “C2609253|Macrovascular disease” exists in the UMLS releases of 2016, MetaMap could not do the mapping.

The column I of the worksheet “VetCN” in the Additional file 1 shows the value “WS” when there is a spelling error. For example, the candidate term “artritis” in row 292 and the candidate term “athritis” in row 321 are incorrect spellings of the term “arthritis”. Candidate terms with wrong spellings are typically not recognised by MetaMap; this is an area for further work.

Aronson and Lang [109] note “*MetaMap’s inability to perform in real-time situations*”. Although this study presents detailed guidelines to decide if a UMLS CUI is a suitable focus concept for an n-gram, it is difficult to envision such guidelines working in real-time without human experts in the loop. Indeed, the SemDeep pipeline presented, whilst delivering promising results for both VetCN and PMSB, is time-consuming and labour-intensive from the domain experts perspective, e.g. four domain experts were needed to validate concept pairs with content from BMJ Best Practice and external resources (when necessary).

This study corroborates the observation made by Pakhomov et al. [60] that word embeddings from the biomedical literature have a performance alike to word embeddings from clinical notes for a semantic similarity and relatedness task. There are two main differences between Pakhomov et al. [60] study and this study:

1. Pakhomov et al. [60] examined only the terms within the medical benchmark dataset [27], which contains 724 single-word pairs. Using single-word terms is a severe limitation as “*most medical terms consist of more than one word*” [60].
2. This study has created neural embeddings from veterinary clinical notes instead of human clinical notes. Hence, this study demonstrated quantitatively the advantages of using a One Health approach instead of keeping the conventional division between veterinary medicine and medical science.

SNOMED CT is the world-leading clinical terminology. In UK, the National Health Service (NHS) has chosen SNOMED CT as the single terminology for the direct management of patient’s care across all care settings in England [110]. A fundamental reason for uptaking a standardised terminology is enabling interoperability [111]. The proposed ontological representation supports:

- Storage of precoordinated SNOMED CT expressions, i.e. UMLS Metathesaurus concepts mapped to SNOMED CT concepts. Hence, this study supports the implementation level 1 (the second level of the three proposed, i.e. 0 to 2) of the SNOMED CT expression storage [112].
- Crafting subsets of SNOMED CT: we also have proposed a step-by-step methodology for SNOMED CT subset development, which has been acknowledged as an unmet need for implementing SNOMED CT in clinical settings [111]. Worldwide, there are three SNOMED CT datasets available to download through the UMLS [14]. In the UK, NHS has developed 80 SNOMED CT human-readable subsets [113]. None of the SNOMED CT datasets mentioned were developed for a well-known medical condition. For example, there is a NHS human-readable subset of SNOMED CT for “Ophthalmology” although not for glaucoma, and likewise, there is a NHS subset for “Respiratory medicine” although not for asthma.
- Building One Health SPARQL SELECT queries that successfully retrieved medical knowledge (validated with BMJ Best Practice content) about the diagnosis and management of the 11 well-known medical conditions that affect humans and animals. These One Health queries exploit knowledge within the locality-based modules extracted from the SNOMED CT ontology and outside of SNOMED CT, i.e. within the OBAN “sometimes true” association relationships.

## Conclusions

In this paper we demonstrated how a Semantic Deep Learning approach can transform neural embeddings of n-grams created from the unstructured text of 300 K PubMed Systematic Reviews (medical science) and 2.5 M veterinary clinical narratives (veterinary medicine) into augmented clinically meaningful biomedical datasets of normalised and interlinked concepts. This study applies the Semantic Web technologies and reuses existing ontologies to 1) encapsulate One Health knowledge about 11 well-known diseases in human and animals that is formal and computable (e.g. allowing a wide range of queries); and 2) separate a “sometimes true” association relationship between two biomedical concepts and its evidence-based provenance (i.e. BMJ Best Practice content). The main benefit of the Semantic Deep Learning approach proposed is in obtaining reliable and usable One Health knowledge (e.g. knowledge useful for public health) that enhances the world-leading clinical terminology SNOMED CT.

## Supplementary information

**Additional file 1.** This file contains 880 term pairs (target term, candidate term) obtained from the two datasets for 11 medical conditions: the worksheet “VetCN” with the 440 term pairs using CBOW and Skip-gram with the VetCN dataset; and the worksheet “PMSB” with the 440 term pairs using CBOW and Skip-gram with the PMSB dataset. Within the worksheet “VetCN” and “PMSB” appear the UMLS CUIs assigned to the candidate terms (n-grams). The worksheet “SF to LF” has the 63 long forms for 80 short forms (including variants of the short forms) within the candidate terms (n-grams). The worksheet “MetaMap performance” contains the number of TP, FP, and FN obtained and used to calculate precision, recall, and F measure for MetaMap in Experiment 1 (EXP-1) and Experiment 2 (EXP-2).

**Additional file 2.** This file contains the guidelines developed for “Step 4: Named entity recognition task”. The file also contains the section “Avoiding pitfalls from the SemDeep pipeline when extracting locality-based modules with SNOMED CT”.

**Additional file 3.** This file shows the results of the evaluation of UMLS CUI pairs with BMJ Best Practice content (i.e. human medicine), i.e. the file contains the 3-tuples (target concept, candidate concept, validation label) for the VetCN dataset (worksheet “VetCN”) and the PMSB dataset (worksheet “PMSB”). The worksheet “signatures” has the ontological signature (i.e. a list of SNOMED CT identifiers) for each of the 11 medical conditions that are the subject of this study. The worksheet “q One Health” shows the number of UMLS CUI pairs validated with BMJ Best Practice content (i.e. human medicine) for each of the 27 UMLS Semantic Types that participates in the SPARQL SELECT query q1VU or q2VU or q3VU (i.e. One Health queries from Table 11).

**Additional file 4.** This file contains the SPARQL SELECT queries; their results appear in Tables 9 and 11.

## Data Availability

All data generated or analysed during this study are included in this article and its Additional files 1,2,3 and 4. This material includes SNOMED Clinical Terms® (SNOMED CT®) which is used by permission of the International Health Terminology Standards Development Organisation (IHTSDO). All rights reserved. SNOMED CT®, was originally created by The College of American Pathologists. “SNOMED” and “SNOMED CT” are registered trademarks of the IHTSDO.

## Abbreviations

ADAM: A database of abbreviations in MEDLINE
ALEHI / ALEI / ALER: A Description Logic (DL) ontology language
Allie: A database and a search service of abbreviations and long forms
API: Application Programming Interface
ARQ: Jena software package
BFO: Basic Formal Ontology
BIBO: Bibliographic Ontology Specification ontology
BMJ: British Medical Journal
BOT: Module type representing bottom modules (aka upper modules)
CBOW: Continuous Bag-of-Words. A neural language model
CHEM: Semantic group “Chemicals & Drugs”
COPD: Chronic Obstructive Pulmonary Disease
CPG: Clinical Practice Guideline
CPU: Central Processing Unit
CUI: Concept Unique Identifier
DL: Description Logic
DynaMed: it publishes clinically-organized topics and provides clinical evidence
ECO: Evidence and Conclusion Ontology
EXP / EXP1 / EXP2: within the paper “EXP” it is a shortened form of “experiment”
FaCT++: it is a tableaux-based reasoner for expressive Description Logics (DL)
FN: False Negative
FP: False Positive
HF: Heart failure
HFpEF: Heart failure with a preserved ejection fraction
IM: within the paper “IM” it is a shortened form of “Incorrectly Mapped”
LDA: Latent Dirichlet Allocation
lemonEXT: within the paper “lemonEXT” it is a shortened form of “extended lemon ontology”
LF: within the paper “LF” it is a shortened form of “long form”
LSA: Latent Semantic Analysis
MEDLINE: a bibliographic database of life sciences and biomedical information
MedlinePlus: a United States of America (USA) National Institutes of Health’s Web site
MeSH: Medical Subject Headings. It is a hierarchically-organized terminology
MetaMap: a tool for recognising UMLS concepts in text
MM: within the paper “MM” it is a shortened form of “Multiple Maps”
MRREL: Related Concepts (File = MRREL.RRF) part of the UMLS (Unified Medical Language System) Metathesaurus
NER: Named-entity recognition
NHS: National Health Service
NICE: the National Institute for Health and Care Excellence
NLP: Natural Language Processing
NM: within the paper “NM” it is a shortened form of “Not Mapped”
NOS: Not Otherwise Specified (or NOS) is a subcategory in systems of disease/ disorder classification
OBAN: Open Biomedical AssociatioNs
OBANmod: within the paper “OBANmod” it is a shortened form of “modified OBAN ontology”
oboInOwl: a meta-model mapping Open Biomedical Ontologies (abbreviated OBO; formerly Open Biological Ontologies) to OWL
Ontolex: Ontology Lexicon ontology
OWL: the Web Ontology Language
PICO: Participants, Interventions, Comparisons and Outcomes
PMSB: within the paper “PMSB” dataset it is a shortened form of “300K PubMed Systematic Review” dataset
PROWESS / PROWESS- SHOCK: randomized clinical trial
PubMed: a free search engine accessing primarily the MEDLINE database
RAM: Random Access Memory
RDF: Resource Description Framework (RDF) format
RO: Relation Ontology
SaRAD: a simple and robust abbreviation dictionary
SAVSNET: Small Animal Veterinary Surveillance Network
SemDeep: Semantic Deep Learning
SF: within the paper “SF” it is a shortened form of “short form”
SF-I: within the paper “SF-I” it is a shortened form of “short form identified incorrectly”
SF-NF: within the paper “SF-NF” it is a shortened form of “not identified” (i.e. “not found”)
Simlex-999 / SimVerb-3500: a benchmark dataset
Skip-gram: a neural language model
SM: within the paper “SM” it is a shortened form of “Single Map”
SNOMED CT: Systematized Nomenclature of Medicine - Clinical Terms. It is a hierarchically-organized terminology
SPARQL: a query language for RDF
TN: True Negative
TP: True Positive
UMLS: Unified Medical Language System
UMLS2016AB: the 2016AB UMLS Metathesaurus release
US / USA: United States of America
USTG: within the paper “USTG” it is a shortened form of “UMLS Semantic Types and Groups”
VetCN: within the paper “VetCN” dataset it is a shortened form of “2.5M veterinary clinical narratives” dataset
VetSCT: the Veterinary Extension for SNOMED CT
