# Supplementary material for: Exploring semantic deep learning for building reliable and reusable one health knowledge from PubMed systematic reviews and veterinary clinical notes
Source: J Biomed Semantics. 2019 Nov 12;10(Suppl 1):22. doi: 10.1186/s13326-019-0212-6 (PMC6849172; doi:10.1186/s13326-019-0212-6)
Supplement: Supplementary file 2 — Additional file 2. This file contains the guidelines developed for “Step 4: Named entity recognition task”. The file also contains the section “Avoiding pitfalls from the SemDeep pipeline when extracting locality-based modules with SNOMED CT”. [file 13326_2019_212_MOESM2_ESM.pdf]

Guidelines developed for Step 4: Named entity recognition task

To determine if MetaMap supplies at least a correct CUI for an n-gram, the following guidelines were developed that intend to favour compatibility with the SNOMED CT Compositional Grammar [1]:

- Single Map (SM) – MetaMap provides a single CUI that captures the full meaning of the n-gram. This case corresponds to "Simple Expression" [1], i.e. a single concept identifier. For example, the n-gram "septic_shock" is only mapped to UMLS CUI = C0036983.
- Multiple Maps (MM) – MetaMap provides multiple CUIs that capture the meaning of the n-gram, and one or more focus concepts may appear among the CUIs provided. This case may also correspond to "Simple Expression" [1], although it more often corresponds to "Expression with Refinements" or "Multiple Focus Concepts" [1]. Selection of the focus concept(s) is guided by six principles:
    1) The focus concept is interpreted in this study as the CUI that captures the key and more specific biomedical/clinical meaning (i.e. governing term) of the n-gram. Therefore, our interpretation of a focus concept is wider than provided by SNOMED CT that interprets a focus concept as "*the part of a SNOMED CT expression that represents a clinical finding, observation, event or procedure*" [2]. In other words, if [1] is strictly followed, a focus concept should belong to the SNOMED CT top-level hierarchy "Clinical finding" or "Observation", or "Event", or "Procedure". For example, the n-gram "procalcitonin-guided_therapy" is mapped to multiple CUIs, where all three "Procalcitonin" and "guided" and "therapy" have CUIs assigned. However, the CUI that captures the key and more specific biomedical/clinical meaning is "C0072027|Procalcitonin", which is mapped in SNOMED CT to "418752001| Procalcitonin (substance)". If [1] is strictly followed, we should not have selected a concept that belongs to the SNOMED CT top-level hierarchy "Substance" as the focus concept. The complete list of SNOMED CT top-level hierarchies appears in [3]. A fundamental reason for interpreting a focus concept that differs from that provided by SNOMED CT is explained below in section "Avoiding pitfalls from the SemDeep pipeline when extracting locality-based modules with SNOMED CT".
    2) When selecting the focus concept, avoid general biomedical/clinical terms in favour of more specific terms. For example, the n-gram "patients_with_severe_sepsis" is mapped to two UMLS Metathesaurus concepts with the highest MetaMap Indexing score (i.e. 1K): the concept "Patients" with UMLS CUI = C0030705; and the concept "Severe Sepsis" with UMLS CUI = C1719672. The focus concept selected should be "C1719672|Severe Sepsis" as it is more specific. This principle also favours selection of "C0072027|Procalcitonin" for the n-gram "procalcitonin-guided_therapy".
    3) When selecting the focus concept, favour CUIs that have a wider coverage in vocabulary sources as well as a wider meaning, and if pertinent, already included in SNOMED CT. For example, the n-gram "neonatal_sepsis" is mapped to two UMLS Metathesaurus concepts with the highest MetaMap Indexing score (i.e. 1K): the concept "Neonatal Sepsis (Sepsis of the newborn)" with UMLS CUI = C0456103; and the concept "Neonatal sepsis (Bacterial sepsis of newborn)" with UMLS CUI = C3665339. Both concepts are included in SNOMED CT. The focus concept selected should be "C0456103|Neonatal Sepsis (Sepsis of the newborn)" as it has a wider coverage in vocabulary sources as well as a wider meaning than "C3665339|Neonatal sepsis (Bacterial sepsis of newborn)".
    4) A focus concept can have one or more refinements (i.e. dependent terms), and thus, this principle supports "Expression with Refinements" [1]. For example, the n-gram "early_goal-directed_therapy" is mapped to two UMLS Metathesaurus concepts: "C1279919|Early" and "C1271494|Goal directed therapy (Goal directed therapy (procedure))". The latter is selected as the focus concept following the first principle and is in agreement with [1] as the UMLS CUI = C1271494 is mapped in SNOMED CT to "391892008|Goal directed therapy (regime/therapy)", which belongs to the SNOMED CT top-level hierarchy "Procedure".
    5) Negation is interpreted in this study as a qualifier, and thus, as a refinement. For example, the n-gram "non-ICU_settings" is mapped to multiple CUIs, where all three "non" and "ICU" and "settings" have CUIs assigned. The focus concept selected should be "C0021708|ICU (intensive care unit)" as it is more specific. Again, our interpretation of a focus concept is wider than that provided by SNOMED CT as "C0021708|ICU (intensive care unit)" is mapped in SNOMED CT to "309904001|Intensive care unit (environment)", which belongs to the SNOMED CT top-level hierarchy "Environment or geographical location".
    6) Multiple focus concepts should be considered only if there is more than one governing term in the n-gram and they belong to the same UMLS Semantic Type. This principle supports "Multiple Focus Concepts" as in [1], which implies concepts from the same SNOMED CT top-level hierarchy. For example, the n-gram "polyclonal intravenous immunoglobulin" is mapped to multiple CUIs: "C0312586|Polyclonal Immunoglobulin (Polyclonal antibody)"; "C0348016|Intravenous"; and "C0085297|Intravenous Immunoglobulin (Immunoglobulins, Intravenous)". The first and last concept can be interpreted as a focus concept as they have in common the UMLS Semantic Types "T116|Amino Acid, Peptide, or Protein" and "T129|Immunologic Factor". However, the two focus concepts selected when mapped to SNOMED CT belong to different SNOMED CT top-level hierarchies: "35310003|Polyclonal antibody (substance)"; and "350344000|Intravenous immunoglobulin (product)". It should be noted that SNOMED CT has distinct concepts for drug classes and ingredients while the UMLS Metathesaurus does not [4].
- Incorrectly Mapped (IM) – MetaMap provides one or more CUIs, however, none captures the meaning of the n-gram. For example, the n-gram "HF" is not mapped to "C0018801|Failure, Heart (Heart failure)".
- Not Mapped (NM) – MetaMap does not provide any CUI. For example, the n-gram "HFpEF" is not mapped to "C3889077|Heart failure with preserved ejection fraction".

Avoiding pitfalls from the SemDeep pipeline when extracting locality-based modules with SNOMED CT

Suppose that "sepsis" is the medical condition under investigation (target term) and we obtain the candidate term (an n-gram among the twenty top-ranked terms with the highest cosine value) "procalcitonin-guided_therapy". If we select as the focus concept "therapy" instead of "procalcitonin" for the n-gram "procalcitonin-guided_therapy", i.e. going against the guidelines introduced in Step 4, the UMLS Metathesaurus concept "C0087111|Therapy (Therapeutic procedure)" will be used for validation with BMJ Best Practice for sepsis [5] as part of the concept pair (C0243026|Sepsis, C0087111|Therapeutic procedure). Every well-known medical condition has a diagnosis and a treatment. Hence, the concept pair (C0243026|Sepsis, C0087111|Therapeutic procedure) will indisputably pass the validation with BMJ Best Practice. Indeed, the term "therapy" appears in BMJ Best Practice for sepsis in a clinically meaningful way considering the following excerpt: "*Diagnostic studies may identify a source of infection that requires removal of a foreign body or drainage to maximise the likelihood of a satisfactory response to therapy*" [5]. Hence, two SNOMED CT concepts that are mapped to the UMLS CUI = "C0087111" would be included in the signature for extracting an upper module from the SNOMED CT ontology about sepsis:

- The SNOMED CT concept "276239002|Therapy (regime/therapy)" with 328 OWL Classes as asserted descendants and 1000 OWL Classes as asserted and inferred descendants using the reasoner FaCT++.
- The SNOMED CT concept "277132007|Therapeutic procedure (procedure)" with 98 OWL Classes as asserted descendants and 2055 OWL Classes as asserted and inferred descendants using the reasoner FaCT++.

Furthermore, if an asserted or inferred descendant of any of these two above-mentioned SNOMED CT concepts has one or more other attribute-value pairs, this will trigger extraction of all axioms that contribute to the meaning of the descendant. There are two undesirable consequences: 1) a significant number of axioms will be extracted; and 2) it is most unlikely that every single descendant of "276239002|Therapy (regime/therapy)" and "277132007|Therapeutic procedure (procedure)" will be semantically related (i.e. "sometimes true" association relationship) to sepsis. Hence, we take an interpretation of a focus concept that differs from the one provided by SNOMED CT (see [1] for details) and favours our aim (stated in the paper subsection "Extracting locality-based modules with SNOMED CT and enabling "One Health" queries" within the section Materials and Method) to extract locality-based modules from the SNOMED CT ontology for well-known medical conditions.

References

1. SNOMED CT Compositional Grammar v2.3.1. http://snomed.org/scg. Accessed 15th May 2018.
2. SNOMED CT: Focus Concept. https://confluence.ihtsdotools.org/display/DOCGLOSS/focus+concept. Accessed 15th May 2018.
3. Systematized Nomenclature of Medicine - Clinical Terms (SNOMED CT). http://snomed.org/sg. Accessed 15th May 2018.
4. SNOMED CT into UMLS Metathesaurus. https://www.nlm.nih.gov/research/umls/Snomed/snomed_represented.html. Accessed 15th May 2018.
5. BMJ Best Practice for sepsis. https://bestpractice.bmj.com/topics/en-gb/245. Accessed 15th May 2018.
