# Supplementary material for: Exploring semantic deep learning for building reliable and reusable one health knowledge from PubMed systematic reviews and veterinary clinical notes
Source: J Biomed Semantics. 2019 Nov 12;10(Suppl 1):22. doi: 10.1186/s13326-019-0212-6 (PMC6849172; doi:10.1186/s13326-019-0212-6)
Supplement: Supplementary file 4 — Additional file 4. This file contains the SPARQL SELECT queries; their results appear in Tables 9 and 11. [file 13326_2019_212_MOESM4_ESM.pdf]

The SPARQL queries for the lemonEXT and OBANmod ontologies: results in Table 9

The following three SPARQL SELECT queries exploit the lemonEXT core ontology once it is populated (the OWL file). The SELECT SPARQL queries q1 and q2 have in bold the UMLS Semantic Types for them. The SELECT SPARQL query q3 has in bold the UMLS Semantic Group to which is applied, i.e. CHEM.

**Query(parameters)**	**SPARQL SELECT Query**
q1(OWLfile,targetTerm)	SELECT (COUNT(DISTINCT ?C) AS ?count)<br>FROM <file:./InOWL/diso11_onto/lemonEXT_core__xmlRdf.owl><br>FROM NAMED <file: OWLfile><br>{ { GRAPH <file: OWLfile> {<br><br>?lx rdf:type lemon:Lexicon ; lemon:entry ?y ; lemon:topic ?targetTerm .<br><br>?targetTerm rdf:type owl:NamedIndividual; rdfs:label ?lbtargetTerm .<br><br>?y rdf:type owl:NamedIndividual; ontolex:denotes ?C .<br><br>?C rdfs:subClassOf ?ST .<br><br>FILTER (**(?ST = ustg:T059 || ?ST = ustg:T060 || ?ST = ustg:T061 || ?ST = ustg:T058)**<br>&& ?lbtargetTerm = "targetTerm"@en)<br>} } }
q2(OWLfile,targetTerm)	SELECT (COUNT(DISTINCT ?C) AS ?count)<br>FROM <file:./InOWL/diso11_onto/lemonEXT_core__xmlRdf.owl><br>FROM NAMED <file: OWLfile><br>{ { GRAPH <file: OWLfile> {<br><br>?lx rdf:type lemon:Lexicon ; lemon:entry ?y ; lemon:topic ?targetTerm .<br><br>?targetTerm rdf:type owl:NamedIndividual; rdfs:label ?lbtargetTerm .<br><br>?y rdf:type owl:NamedIndividual; ontolex:denotes ?C .<br><br>?C rdfs:subClassOf ?ST .<br><br>FILTER (**(?ST = ustg:T034 || ?ST = ustg:T184 || ?ST = ustg:T033)**<br>&& ?lbtargetTerm = "targetTerm"@en)<br>} } }
q3(OWLfile,targetTerm)	SELECT (COUNT(DISTINCT ?C) AS ?count)<br>FROM <file:./InOWL/diso11_onto/lemonEXT_core__xmlRdf.owl><br>FROM NAMED <file: OWLfile><br>{ { GRAPH <file: OWLfile> {<br><br>?lx rdf:type lemon:Lexicon ; lemon:entry ?y ; lemon:topic ?targetTerm .<br><br>?targetTerm rdf:type owl:NamedIndividual; rdfs:label ?lbtargetTerm .<br><br>?y rdf:type owl:NamedIndividual; ontolex:denotes ?C .<br><br>?C rdfs:subClassOf ?ST .<br><br>?ST rdf:type owl:Class ; rdfs:subClassOf [a owl:Restriction ;<br>owl:onProperty obo:BFO_0000050; owl:someValuesFrom ?SG] .<br><br>FILTER (**?SG = ustg:CHEM** && ?lbtargetTerm = "targetTerm"@en)<br>} } }

The next three SPARQL SELECT queries exploit the OBANmod core ontology once it is populated (the OWL file). The SELECT SPARQL queries q1V and q2V have in bold the UMLS Semantic Types for them. The SELECT SPARQL query q3V has in bold the UMLS Semantic Group to which is applied, i.e. CHEM.

<table>
<tr><th>Query(parameters)</th><th>SPARQL SELECT Query</th></tr>
<tr><td>q1V(OWLfile,CUItargetTerm)</td><td>SELECT (COUNT(DISTINCT ?C) AS ?count)
FROM <file:./InOWL/diso11_onto/OBANmod_core__xmlRdf.owl>
FROM NAMED <file:OWLfile>
{ { GRAPH <file:OWLfile> {

?x rdf:type oban:association ; oban:association_has_object ?C ;
oban:association_has_subject ustg:CUItargetTerm .

?C rdfs:subClassOf ?ST .

?y rdf:type oban:provenance ; obo:IAO_0000136 ?x ; obo:RO_0002558 ?lbV .

FILTER (**(?ST = ustg:T059 || ?ST = ustg:T060 || ?ST = ustg:T061 ||
?ST = ustg:T058)** &&
(?lbV = OBANmod:OBANmod_2 || ?lbV = OBANmod:OBANmod_3 ||
?lbV = OBANmod:OBANmod_4 || ?lbV = OBANmod:OBANmod_5))
} } }</td></tr>
<tr><td>q2V(OWLfile, CUItargetTerm)</td><td>SELECT (COUNT(DISTINCT ?C) AS ?count)
FROM <file:./InOWL/diso11_onto/OBANmod_core__xmlRdf.owl>
FROM NAMED <file:OWLfile>
{ { GRAPH <file:OWLfile> {

?x rdf:type oban:association ; oban:association_has_object ?C ;
oban:association_has_subject ustg:CUItargetTerm .

?C rdfs:subClassOf ?ST .

?y rdf:type oban:provenance ; obo:IAO_0000136 ?x ; obo:RO_0002558 ?lbV .

FILTER (**(?ST = ustg:T034 || ?ST = ustg:T184 || ?ST = ustg:T033)** &&
(?lbV = OBANmod:OBANmod_2 || ?lbV = OBANmod:OBANmod_3 ||
?lbV = OBANmod:OBANmod_4 || ?lbV = OBANmod:OBANmod_5))
} } }</td></tr>
<tr><td>q3V(OWLfile, CUItargetTerm)</td><td>SELECT (COUNT(DISTINCT ?C) AS ?count)
FROM <file:./InOWL/diso11_onto/OBANmod_core__xmlRdf.owl>
FROM NAMED <file:OWLfile>
{ { GRAPH <file:OWLfile> {

?x rdf:type oban:association ; oban:association_has_object ?C ;
oban:association_has_subject ustg:CUItargetTerm .

?C rdfs:subClassOf ?ST .

?ST rdf:type owl:Class ; rdfs:subClassOf [a owl:Restriction ;
owl:onProperty obo:BFO_0000050; owl:someValuesFrom ?SG] .

?y rdf:type oban:provenance ; obo:IAO_0000136 ?x ; obo:RO_0002558 ?lbV .

FILTER (**?SG = ustg:CHEM** &&
(?lbV = OBANmod:OBANmod_2 || ?lbV = OBANmod:OBANmod_3 ||
?lbV = OBANmod:OBANmod_4 || ?lbV = OBANmod:OBANmod_5))
} } }</td></tr>
</table>

“One Health” queries in SPARQL: results in Table 11

The following three SPARQL SELECT queries exploit the OBANmod core ontology once it is populated for both datasets, i.e. the VetCN dataset (the OWL file 1) and the PMSB dataset (the OWL file 2). The SELECT SPARQL queries q1VU and q2VU have in bold the UMLS Semantic Types for them. The SELECT SPARQL query q3VU has in bold the UMLS Semantic Group to which is applied, i.e. CHEM.

Query(parameters)	SPARQL SELECT Query
q1VU(OWLfile1, OWLfile2, CUItargetTerm)	SELECT (COUNT(DISTINCT ?C) AS ?count)<br>FROM <file:./InOWL/diso11_onto/OBANmod_core__xmlRdf.owl><br>FROM NAMED <file:OWLfile1><br>FROM NAMED <file:OWLfile2><br>{ { GRAPH <file:OWLfile1> {<br><br>?x rdf:type oban:association ; oban:association_has_object ?C ;<br>oban:association_has_subject ustg:CUItargetTerm .<br><br>?C rdfs:subClassOf ?ST .<br><br>?y rdf:type oban:provenance ; obo:IAO_0000136 ?x ; obo:RO_0002558 ?lbV .<br><br>FILTER (**(?ST = ustg:T059 || ?ST = ustg:T060 || ?ST = ustg:T061 || ?ST = ustg:T058)**<br>&& (?lbV = OBANmod:OBANmod_2 || ?lbV = OBANmod:OBANmod_3 || ?lbV = OBANmod:OBANmod_4 || ?lbV = OBANmod:OBANmod_5)) } }<br><br>UNION<br><br>{ GRAPH <file:OWLfile2 > {<br><br>?x rdf:type oban:association ; oban:association_has_object ?C ;<br>oban:association_has_subject ustg:CUItargetTerm .<br><br>?C rdfs:subClassOf ?ST .<br><br>?y rdf:type oban:provenance ; obo:IAO_0000136 ?x ; obo:RO_0002558 ?lbV .<br><br>FILTER (**(?ST = ustg:T059 || ?ST = ustg:T060 || ?ST = ustg:T061 || ?ST = ustg:T058)**<br>&& (?lbV = OBANmod:OBANmod_2 || ?lbV = OBANmod:OBANmod_3 || ?lbV = OBANmod:OBANmod_4 || ?lbV = OBANmod:OBANmod_5))<br>} } }
q2VU(OWLfile1, OWLfile2, CUItargetTerm)	SELECT (COUNT(DISTINCT ?C) AS ?count)<br>FROM <file:./InOWL/diso11_onto/OBANmod_core__xmlRdf.owl><br>FROM NAMED <file:OWLfile1><br>FROM NAMED <file:OWLfile2><br>{ { GRAPH <file:OWLfile1> {<br><br>?x rdf:type oban:association ; oban:association_has_object ?C ;<br>oban:association_has_subject ustg:CUItargetTerm .<br><br>?C rdfs:subClassOf ?ST .<br><br>?y rdf:type oban:provenance ; obo:IAO_0000136 ?x ; obo:RO_0002558 ?lbV .<br><br>FILTER (**(?ST = ustg:T034 || ?ST = ustg:T184 || ?ST = ustg:T033)**<br>&& (?lbV = OBANmod:OBANmod_2 || ?lbV = OBANmod:OBANmod_3 || ?lbV = OBANmod:OBANmod_4 || ?lbV = OBANmod:OBANmod_5)) } }<br><br>UNION

<table>
<tr><td></td><td>{ GRAPH <file:OWLfile2 > {

?x rdf:type oban:association ; oban:association_has_object ?C ;
oban:association_has_subject ustg:CUItargetTerm .

?C rdfs:subClassOf ?ST .

?y rdf:type oban:provenance ; obo:IAO_0000136 ?x ; obo:RO_0002558 ?lbV .

FILTER ((?ST = ustg:T034 || ?ST = ustg:T184 || ?ST = ustg:T033)
&& (?lbV = OBANmod:OBANmod_2 || ?lbV = OBANmod:OBANmod_3 ||
?lbV = OBANmod:OBANmod_4 || ?lbV = OBANmod:OBANmod_5))
} } }</td></tr>
<tr><td>q3VU(OWLfile1, OWLfile2, CUItargetTerm)</td><td>SELECT (COUNT(DISTINCT ?C) AS ?count)
FROM <file:./InOWL/diso11_onto/OBANmod_core__xmlRdf.owl>
FROM NAMED <file:OWLfile1>
FROM NAMED <file:OWLfile2>
{ { GRAPH <file:OWLfile1> {

?x rdf:type oban:association ; oban:association_has_object ?C ;
oban:association_has_subject ustg:CUItargetTerm .

?C rdfs:subClassOf ?ST .

?ST rdf:type owl:Class ; rdfs:subClassOf [a owl:Restriction ;
owl:onProperty obo:BFO_0000050; owl:someValuesFrom ?SG] .

?y rdf:type oban:provenance ; obo:IAO_0000136 ?x ; obo:RO_0002558 ?lbV .

FILTER (?SG = ustg:CHEM
&& (?lbV = OBANmod:OBANmod_2 || ?lbV = OBANmod:OBANmod_3 ||
?lbV = OBANmod:OBANmod_4 || ?lbV = OBANmod:OBANmod_5)) } }

UNION

{ GRAPH <file:OWLfile2 > {

?x rdf:type oban:association ; oban:association_has_object ?C ;
oban:association_has_subject ustg:CUItargetTerm .

?C rdfs:subClassOf ?ST .

?ST rdf:type owl:Class ; rdfs:subClassOf [a owl:Restriction ;
owl:onProperty obo:BFO_0000050; owl:someValuesFrom ?SG] .

?y rdf:type oban:provenance ; obo:IAO_0000136 ?x ; obo:RO_0002558 ?lbV .

FILTER (?SG = ustg:CHEM
&& (?lbV = OBANmod:OBANmod_2 || ?lbV = OBANmod:OBANmod_3 ||
?lbV = OBANmod:OBANmod_4 || ?lbV = OBANmod:OBANmod_5))
} } }</td></tr>
</table>

The following three SPARQL SELECT queries exploit the OBANmod core ontology once it is populated for both datasets, i.e. the VetCN dataset (the OWL file 1) and the PMSB dataset (the OWL file 2). The SELECT SPARQL queries q1VM and q2VM have in bold the UMLS Semantic Types for them. The SELECT SPARQL query q3VM has in bold the UMLS Semantic Group to which is applied, i.e. CHEM.

<table>
<tr><th>Query(parameters)</th><th>SPARQL SELECT Query</th></tr>
<tr><td>q1VM(OWLfile1,
OWLfile2,
CUItargetTerm)</td><td>SELECT (COUNT(DISTINCT ?C) AS ?count)
FROM <file:./InOWL/diso11_onto/OBANmod_core__xmlRdf.owl>
FROM NAMED <file:OWLfile1>
FROM NAMED <file:OWLfile2>
{ { GRAPH <file:OWLfile1> {

?x rdf:type oban:association ; oban:association_has_object ?C ;
oban:association_has_subject ustg:CUItargetTerm .

?C rdfs:subClassOf ?ST ; ustg:hasDbXrefInSCT ?SCTid .

?y rdf:type oban:provenance ; obo:IAO_0000136 ?x ; obo:RO_0002558 ?lbV .

FILTER (**(?ST = ustg:T059 || ?ST = ustg:T060 || ?ST = ustg:T061 || ?ST = ustg:T058)**
&& (?lbV = OBANmod:OBANmod_2 || ?lbV = OBANmod:OBANmod_3 ||
?lbV = OBANmod:OBANmod_4 || ?lbV = OBANmod:OBANmod_5)) } }

UNION

{ GRAPH <file:OWLfile2 > {

?x rdf:type oban:association ; oban:association_has_object ?C ;
oban:association_has_subject ustg:CUItargetTerm .

?C rdfs:subClassOf ?ST ; ustg:hasDbXrefInSCT ?SCTid .

?y rdf:type oban:provenance ; obo:IAO_0000136 ?x ; obo:RO_0002558 ?lbV .

FILTER (**(?ST = ustg:T059 || ?ST = ustg:T060 || ?ST = ustg:T061 || ?ST = ustg:T058)**
&& (?lbV = OBANmod:OBANmod_2 || ?lbV = OBANmod:OBANmod_3 ||
?lbV = OBANmod:OBANmod_4 || ?lbV = OBANmod:OBANmod_5))
} } }</td></tr>
<tr><td>q2VM(OWLfile1,
OWLfile2,
CUItargetTerm)</td><td>SELECT (COUNT(DISTINCT ?C) AS ?count)
FROM <file:./InOWL/diso11_onto/OBANmod_core__xmlRdf.owl>
FROM NAMED <file:OWLfile1>
FROM NAMED <file:OWLfile2>
{ { GRAPH <file:OWLfile1> {

?x rdf:type oban:association ; oban:association_has_object ?C ;
oban:association_has_subject ustg:CUItargetTerm .

?C rdfs:subClassOf ?ST ; ustg:hasDbXrefInSCT ?SCTid .

?y rdf:type oban:provenance ; obo:IAO_0000136 ?x ; obo:RO_0002558 ?lbV .

FILTER (**(?ST = ustg:T034 || ?ST = ustg:T184 || ?ST = ustg:T033)**
&& (?lbV = OBANmod:OBANmod_2 || ?lbV = OBANmod:OBANmod_3 ||
?lbV = OBANmod:OBANmod_4 || ?lbV = OBANmod:OBANmod_5))
} }

UNION</td></tr>
</table>

<table>
<tr><td></td><td>{ GRAPH <file:OWLfile2 > {

?x rdf:type oban:association ; oban:association_has_object ?C ;
    oban:association_has_subject ustg:CUItargetTerm .

?C rdfs:subClassOf ?ST ; ustg:hasDbXrefInSCT ?SCTid .

?y rdf:type oban:provenance ; obo:IAO_0000136 ?x ; obo:RO_0002558 ?lbV .

 FILTER ((?ST = ustg:T034 || ?ST = ustg:T184 || ?ST = ustg:T033)
    && (?lbV = OBANmod:OBANmod_2 || ?lbV = OBANmod:OBANmod_3 ||
    ?lbV = OBANmod:OBANmod_4 || ?lbV = OBANmod:OBANmod_5))
} } }</td></tr>
<tr><td>q3VM(OWLfile1, OWLfile2, CUItargetTerm)</td><td>SELECT (COUNT(DISTINCT ?C) AS ?count)
FROM <file:./InOWL/diso11_onto/OBANmod_core__xmlRdf.owl>
FROM NAMED <file:OWLfile1>
FROM NAMED <file:OWLfile2>
 { { GRAPH <file:OWLfile1> {

?x rdf:type oban:association ; oban:association_has_object ?C ;
    oban:association_has_subject ustg:CUItargetTerm .

 ?C rdfs:subClassOf ?ST ; ustg:hasDbXrefInSCT ?SCTid .

?ST rdf:type owl:Class ; rdfs:subClassOf [a owl:Restriction ;
          owl:onProperty obo:BFO_0000050; owl:someValuesFrom ?SG] .

?y rdf:type oban:provenance ; obo:IAO_0000136 ?x ; obo:RO_0002558 ?lbV .

 FILTER (?SG = ustg:CHEM
     && (?lbV = OBANmod:OBANmod_2 || ?lbV = OBANmod:OBANmod_3 ||
     ?lbV = OBANmod:OBANmod_4 || ?lbV = OBANmod:OBANmod_5)) } }

 UNION

{ GRAPH <file:OWLfile2 > {

?x rdf:type oban:association ; oban:association_has_object ?C ;
    oban:association_has_subject ustg:CUItargetTerm .

?C rdfs:subClassOf ?ST ; ustg:hasDbXrefInSCT ?SCTid .

?ST rdf:type owl:Class ; rdfs:subClassOf [a owl:Restriction ;
          owl:onProperty obo:BFO_0000050; owl:someValuesFrom ?SG] .

?y rdf:type oban:provenance ; obo:IAO_0000136 ?x ; obo:RO_0002558 ?lbV .

 FILTER (?SG = ustg:CHEM
     && (?lbV = OBANmod:OBANmod_2 || ?lbV = OBANmod:OBANmod_3 ||
    ?lbV = OBANmod:OBANmod_4 || ?lbV = OBANmod:OBANmod_5))
} } }</td></tr>
</table>

The following three SPARQL SELECT queries exploit: a) the asserted information within the locality-based modules created per target term from the SNOMED CT ontology; and b) the OBANmod core ontology once it is populated for both datasets, i.e. the VetCN dataset (the OWL file 1) and the PMSB dataset (the OWL file 2). The SELECT SPARQL queries q1VS and q2VS have in bold the UMLS Semantic Types for them. The SELECT SPARQL query q3VS has in bold the UMLS Semantic Group to which is applied, i.e. CHEM.

**Query(parameters)**	**SPARQL SELECT Query**
q1VS(OWLfile1,<br>OWLfile2,<br>CUItargetTerm,<br>OWLtargetTermModule)	SELECT (COUNT(DISTINCT ?SCT) AS ?count)<br>FROM <file:OWLtargetTermModule><br>FROM NAMED <file:OWLfile1><br>FROM NAMED <file:OWLfile2><br>{ { GRAPH <file:OWLfile1> {<br><br>?x rdf:type oban:association ; oban:association_has_object ?C ;<br>oban:association_has_subject ustg:CUItargetTerm .<br><br>?C rdfs:subClassOf ?ST ; ustg:hasDbXrefInSCT ?SCTid .<br><br>?y rdf:type oban:provenance ; obo:IAO_0000136 ?x ; obo:RO_0002558 ?lbV .<br><br>FILTER (**(?ST = ustg:T059** || **?ST = ustg:T060** || **?ST = ustg:T061** || **?ST = ustg:T058)**<br>&& (?lbV = OBANmod:OBANmod_2 || ?lbV = OBANmod:OBANmod_3 || ?lbV = OBANmod:OBANmod_4 || ?lbV = OBANmod:OBANmod_5))<br>}<br><br>?SCT a owl:Class .<br><br>FILTER (fn:ends-with(xsd:string(?SCT), "/" + ?SCTid))<br><br>}<br><br>UNION<br><br>{ GRAPH <file:OWLfile2 > {<br><br>?x rdf:type oban:association ; oban:association_has_object ?C ;<br>oban:association_has_subject ustg:CUItargetTerm .<br><br>?C rdfs:subClassOf ?ST ; ustg:hasDbXrefInSCT ?SCTid .<br><br>?y rdf:type oban:provenance ; obo:IAO_0000136 ?x ; obo:RO_0002558 ?lbV .<br><br>FILTER (**(?ST = ustg:T059** || **?ST = ustg:T060** || **?ST = ustg:T061** || **?ST = ustg:T058)**<br>&& (?lbV = OBANmod:OBANmod_2 || ?lbV = OBANmod:OBANmod_3 || ?lbV = OBANmod:OBANmod_4 || ?lbV = OBANmod:OBANmod_5))<br>}<br><br>?SCT a owl:Class .<br><br>FILTER (fn:ends-with(xsd:string(?SCT), "/" + ?SCTid))<br><br>} } }

q2VS(OWLfile1,<br>OWLfile2,<br>CUItargetTerm,<br>OWLtargetTermModule)	SELECT (COUNT(DISTINCT ?SCT) AS ?count)<br>FROM <file:OWLtargetTermModule><br>FROM NAMED <file:OWLfile1><br>FROM NAMED <file:OWLfile2><br>{ { GRAPH <file:OWLfile1> {<br><br>?x rdf:type oban:association ; oban:association_has_object ?C ;<br>oban:association_has_subject ustg:CUItargetTerm .<br><br>?C rdfs:subClassOf ?ST ; ustg:hasDbXrefInSCT ?SCTid .<br><br>?y rdf:type oban:provenance ; obo:IAO_0000136 ?x ; obo:RO_0002558 ?lbV .<br><br>FILTER (**(?ST = ustg:T034 || ?ST = ustg:T184 || ?ST = ustg:T033)**<br>&& (?lbV = OBANmod:OBANmod_2 || ?lbV = OBANmod:OBANmod_3 || ?lbV = OBANmod:OBANmod_4 || ?lbV = OBANmod:OBANmod_5))<br>}<br><br>?SCT a owl:Class .<br><br>FILTER (fn:ends-with(xsd:string(?SCT), "/" + ?SCTid))<br><br>}<br><br>UNION<br><br>{ GRAPH <file:OWLfile2 > {<br><br>?x rdf:type oban:association ; oban:association_has_object ?C ;<br>oban:association_has_subject ustg:CUItargetTerm .<br><br>?C rdfs:subClassOf ?ST ; ustg:hasDbXrefInSCT ?SCTid .<br><br>?y rdf:type oban:provenance ; obo:IAO_0000136 ?x ; obo:RO_0002558 ?lbV .<br><br>FILTER (**(?ST = ustg:T034 || ?ST = ustg:T184 || ?ST = ustg:T033)**<br>&& (?lbV = OBANmod:OBANmod_2 || ?lbV = OBANmod:OBANmod_3 || ?lbV = OBANmod:OBANmod_4 || ?lbV = OBANmod:OBANmod_5))<br><br>}<br><br>?SCT a owl:Class .<br><br>FILTER (fn:ends-with(xsd:string(?SCT), "/" + ?SCTid))<br><br>} }

q3VS(OWLfile1, OWLfile2, CUItargetTerm, OWLtargetTermModule)	see query below

```
SELECT (COUNT(DISTINCT ?SCT) AS ?count)
FROM <file:OWLtargetTermModule>
FROM NAMED <file:OWLfile1>
FROM NAMED <file:OWLfile2>
 { {  GRAPH <file:OWLfile1> {

?x rdf:type oban:association ; oban:association_has_object ?C ;
    oban:association_has_subject  ustg:CUItargetTerm .

 ?C rdfs:subClassOf ?ST ;  ustg:hasDbXrefInSCT  ?SCTid .

?ST rdf:type owl:Class ; rdfs:subClassOf  [ a owl:Restriction ;
          owl:onProperty obo:BFO_0000050; owl:someValuesFrom ?SG  ] .

?y rdf:type oban:provenance ; obo:IAO_0000136 ?x ; obo:RO_0002558 ?lbV .

 FILTER (  ?SG = ustg:CHEM
     && (?lbV  = OBANmod:OBANmod_2 || ?lbV  = OBANmod:OBANmod_3 ||
     ?lbV  = OBANmod:OBANmod_4 || ?lbV  = OBANmod:OBANmod_5 ) )

}

  ?SCT a owl:Class .

  FILTER ( fn:ends-with(xsd:string(?SCT), "/" + ?SCTid) )

}

 UNION

{  GRAPH <file:OWLfile2 > {

?x rdf:type oban:association ; oban:association_has_object ?C ;
    oban:association_has_subject  ustg:CUItargetTerm .

 ?C rdfs:subClassOf ?ST ;  ustg:hasDbXrefInSCT  ?SCTid .

?ST rdf:type owl:Class ; rdfs:subClassOf  [ a owl:Restriction ;
          owl:onProperty obo:BFO_0000050; owl:someValuesFrom ?SG  ] .

?y rdf:type oban:provenance ; obo:IAO_0000136 ?x ; obo:RO_0002558 ?lbV .

 FILTER ( ?SG = ustg:CHEM
     && (?lbV  = OBANmod:OBANmod_2 || ?lbV  = OBANmod:OBANmod_3 ||
    ?lbV  = OBANmod:OBANmod_4 || ?lbV  = OBANmod:OBANmod_5 ) )

}

  ?SCT a owl:Class .

  FILTER ( fn:ends-with(xsd:string(?SCT), "/" + ?SCTid) )

} }
```

The following three SPARQL SELECT queries exploit: a) the inferred information obtained (with the reasoner FaCT++) from the axioms within the locality-based modules created per target term from the SNOMED CT ontology; and b) the OBANmod core ontology once it is populated for both datasets, i.e. the VetCN dataset (the OWL file 1) and the PMSB dataset (the OWL file 2). The SELECT SPARQL queries q1VR and q2VR have in bold the UMLS Semantic Types for them. The SELECT SPARQL query q3VR has in bold the UMLS Semantic Group to which is applied, i.e. CHEM.

<table>
<tr><th>Query(parameters)</th><th>SPARQL SELECT Query</th></tr>
<tr><td>q1VR(OWLfile1, OWLfile2, CUItargetTerm, inferred_ OWLtargetTermModule)</td><td>SELECT (COUNT(DISTINCT ?SCTdes) AS ?count)
FROM <file:inferredOWLtargetTermModule>
FROM NAMED <file:OWLfile1>
FROM NAMED <file:OWLfile2>
{ { GRAPH <file:OWLfile1> {

?x rdf:type oban:association ; oban:association_has_object ?C ;
oban:association_has_subject ustg:CUItargetTerm .

?C rdfs:subClassOf ?ST ; ustg:hasDbXrefInSCT ?SCTid .

?y rdf:type oban:provenance ; obo:IAO_0000136 ?x ; obo:RO_0002558 ?lbV .

FILTER ((?ST = ustg:T059 || ?ST = ustg:T060 || ?ST = ustg:T061 || ?ST = ustg:T058)
&& (?lbV = OBANmod:OBANmod_2 || ?lbV = OBANmod:OBANmod_3 \|\| ?lbV = OBANmod:OBANmod_4 || ?lbV = OBANmod:OBANmod_5))
}

?SCTdes rdfs:subClassOf+ ?SCT .

FILTER (fn:ends-with(xsd:string(?SCT), "/" + ?SCTid))

}

UNION

{ GRAPH <file:OWLfile2 > {

?x rdf:type oban:association ; oban:association_has_object ?C ;
oban:association_has_subject ustg:CUItargetTerm .

?C rdfs:subClassOf ?ST ;ustg:hasDbXrefInSCT ?SCTid .

?y rdf:type oban:provenance ; obo:IAO_0000136 ?x ; obo:RO_0002558 ?lbV .

FILTER ((?ST = ustg:T059 || ?ST = ustg:T060 || ?ST = ustg:T061 || ?ST = ustg:T058)
&& (?lbV = OBANmod:OBANmod_2 || ?lbV = OBANmod:OBANmod_3 || ?lbV = OBANmod:OBANmod_4 || ?lbV = OBANmod:OBANmod_5))
}

?SCTdes rdfs:subClassOf+ ?SCT .

FILTER (fn:ends-with(xsd:string(?SCT), "/" + ?SCTid))

} } }</td></tr>
</table>

q2VR(OWLfile1, OWLfile2, CUItargetTerm, inferred_ OWLtargetTermModule)	SELECT (COUNT(DISTINCT ?SCTdes) AS ?count)<br>FROM <file:inferredOWLtargetTermModule><br>FROM NAMED <file:OWLfile1><br>FROM NAMED <file:OWLfile2><br>{ { GRAPH <file:OWLfile1> {<br><br>?x rdf:type oban:association ; oban:association_has_object ?C ;<br>oban:association_has_subject ustg:CUItargetTerm .<br><br>?C rdfs:subClassOf ?ST ; ustg:hasDbXrefInSCT ?SCTid .<br><br>?y rdf:type oban:provenance ; obo:IAO_0000136 ?x ; obo:RO_0002558 ?lbV .<br><br>FILTER (**(?ST = ustg:T034 || ?ST = ustg:T184 || ?ST = ustg:T033)**<br>&& (?lbV = OBANmod:OBANmod_2 || ?lbV = OBANmod:OBANmod_3 || ?lbV = OBANmod:OBANmod_4 || ?lbV = OBANmod:OBANmod_5))<br>}<br><br>?SCTdes rdfs:subClassOf+ ?SCT .<br><br>FILTER (fn:ends-with(xsd:string(?SCT), "/" + ?SCTid))<br><br>}<br><br>UNION<br><br>{ GRAPH <file:OWLfile2 > {<br><br>?x rdf:type oban:association ; oban:association_has_object ?C ;<br>oban:association_has_subject ustg:CUItargetTerm .<br><br>?C rdfs:subClassOf ?ST ; ustg:hasDbXrefInSCT ?SCTid .<br><br>?y rdf:type oban:provenance ; obo:IAO_0000136 ?x ; obo:RO_0002558 ?lbV .<br><br>FILTER (**(?ST = ustg:T034 || ?ST = ustg:T184 || ?ST = ustg:T033)**<br>&& (?lbV = OBANmod:OBANmod_2 || ?lbV = OBANmod:OBANmod_3 || ?lbV = OBANmod:OBANmod_4 || ?lbV = OBANmod:OBANmod_5))<br><br>}<br><br>?SCTdes rdfs:subClassOf+ ?SCT .<br><br>FILTER (fn:ends-with(xsd:string(?SCT), "/" + ?SCTid))<br><br>} }

<table>
<tr>
<td>q3VR(OWLfile1, OWLfile2, CUItargetTerm, inferred_ OWLtargetTermModule)</td>
<td>SELECT (COUNT(DISTINCT ?SCTdes) AS ?count)
FROM <file:inferredOWLtargetTermModule>
FROM NAMED <file:OWLfile1>
FROM NAMED <file:OWLfile2>
{ { GRAPH <file:OWLfile1> {

?x rdf:type oban:association ; oban:association_has_object ?C ;
oban:association_has_subject ustg:CUItargetTerm .

?C rdfs:subClassOf ?ST ; ustg:hasDbXrefInSCT ?SCTid .

?ST rdf:type owl:Class ; rdfs:subClassOf [a owl:Restriction ;
owl:onProperty obo:BFO_0000050; owl:someValuesFrom ?SG] .

?y rdf:type oban:provenance ; obo:IAO_0000136 ?x ; obo:RO_0002558 ?lbV .

FILTER (?SG = ustg:CHEM
&& (?lbV = OBANmod:OBANmod_2 || ?lbV = OBANmod:OBANmod_3 ||
?lbV = OBANmod:OBANmod_4 || ?lbV = OBANmod:OBANmod_5))

}

?SCTdes rdfs:subClassOf+ ?SCT .

FILTER (fn:ends-with(xsd:string(?SCT), "/" + ?SCTid))

}

UNION

{ GRAPH <file:OWLfile2 > {

?x rdf:type oban:association ; oban:association_has_object ?C ;
oban:association_has_subject ustg:CUItargetTerm .

?C rdfs:subClassOf ?ST ; ustg:hasDbXrefInSCT ?SCTid .

?ST rdf:type owl:Class ; rdfs:subClassOf [a owl:Restriction ;
owl:onProperty obo:BFO_0000050; owl:someValuesFrom ?SG] .

?y rdf:type oban:provenance ; obo:IAO_0000136 ?x ; obo:RO_0002558 ?lbV .

FILTER (?SG = ustg:CHEM
&& (?lbV = OBANmod:OBANmod_2 || ?lbV = OBANmod:OBANmod_3 ||
?lbV = OBANmod:OBANmod_4 || ?lbV = OBANmod:OBANmod_5))

}

?SCTdes rdfs:subClassOf+ ?SCT .

FILTER (fn:ends-with(xsd:string(?SCT), "/" + ?SCTid))

} }</td>
</tr>
</table>
